# Androgen responsive intronic non-coding RNAs

**DOI:** 10.1186/1741-7007-5-4

**Published:** 2007-01-30

**Authors:** Rodrigo Louro, Helder I Nakaya, Paulo P Amaral, Fernanda Festa, Mari C Sogayar, Aline M da Silva, Sergio Verjovski-Almeida, Eduardo M Reis

**Affiliations:** 1 Departamento de Bioquimica, Instituto de Quimica, Universidade de São Paulo, 05508-900 São Paulo, Brazil

## Abstract

**Background:**

Transcription of large numbers of non-coding RNAs originating from intronic regions of human genes has been recently reported, but mechanisms governing their biosynthesis and biological functions are largely unknown. In this work, we evaluated the existence of a common mechanism of transcription regulation shared by protein-coding mRNAs and intronic RNAs by measuring the effect of androgen on the transcriptional profile of a prostate cancer cell line.

**Results:**

Using a custom-built cDNA microarray enriched in intronic transcribed sequences, we found 39 intronic non-coding RNAs for which levels were significantly regulated by androgen exposure. Orientation-specific reverse transcription-PCR indicated that 10 of the 13 were transcribed in the antisense direction. These transcripts are long (0.5–5 kb), unspliced and apparently do not code for proteins. Interestingly, we found that the relative levels of androgen-regulated intronic transcripts could be correlated with the levels of the corresponding protein-coding gene (*asGAS6 *and *asDNAJC3*) or with the alternative usage of exons (*asKDELR2 *and *asITGA6*) in the corresponding protein-coding transcripts. Binding of the androgen receptor to a putative regulatory region upstream from *asMYO5A*, an androgen-regulated antisense intronic transcript, was confirmed by chromatin immunoprecipitation.

**Conclusion:**

Altogether, these results indicate that at least a fraction of naturally transcribed intronic non-coding RNAs may be regulated by common physiological signals such as hormones, and further corroborate the notion that the intronic complement of the transcriptome play functional roles in the human gene-expression program.

## Background

Non-coding RNAs (ncRNAs), particularly those transcribed from genomic regions spanning introns of spliced genes, have been proposed as a fundamental advance in the genetic operating system of higher organisms by influencing the genomic program of differentiation and development in individuals and species [1,2]. Many functions, such as transcriptional or translational regulation, RNA splicing, gene silencing, imprinting and dosage compensation, have been assigned to mammalian ncRNAs [3]. Most functionally characterized ncRNAs belong to classes of small RNAs such as microRNAs [4] and small nucleolar RNAs (snoRNAs) [5]. While several novel long ncRNAs have been described in mammalian organisms [6,7] few have been characterized in more detail, the ~15 kb Xist RNA involved in inactivation of chromosome X being an exception [8].

Recent studies based on computational analysis and experimental validation have revealed that both introns and intergenic regions constitute major sources of ncRNAs [3,9,10]. Extensive analyses of the human expressed sequence tags (EST) database have focused particularly on spliced mRNAs, and ESTs that overlap at least one exon have identified an abundant number of sense-antisense transcript pairs in humans [7,11,12], mice [13] and plants [14], pointing to a well-conserved mechanism of post-transcriptional regulation of gene expression in eukaryotes.

Recently, microRNAs have been shown to be consistently altered in normal and tumor cells, allowing the identification of tissue-specific expression signatures [15]. Using a custom-built cDNA microarray enriched in ESTs corresponding to fragments of intronic transcripts, we previously detected transcription of a set of totally intronic ncRNAs, which are long (0.6–1.1 Kb), unspliced, and oriented in the antisense direction relative to the corresponding protein-coding transcript [16]. Moreover, expression levels of 23 intronic ncRNAs were shown to correlate with the degree of tumor differentiation in prostate cancer [16]. The potential role of long ncRNAs in human cancer is also exemplified by *MALAT-1*, a 7-kb RNA that was reported to be associated with metastatic non-small cell lung cancer [17]. Other reports have shown that totally or partially intronic long antisense transcripts may have regulatory functions, such as modulating the methylation status of promoters [18] or the alternative splicing pattern of the corresponding protein-coding gene [19]. However, little attention has been given to the mechanism used by the cell to control the expression of these long intronic RNAs.

Androgens have been implicated in central events governing the regulation of distinct and diverse physiological processes in normal and neoplastic prostate cells. These hormones are known to promote cell division and proliferation of epithelial cells, to modulate programmed cell death and cell quiescence [20,21] and to regulate cellular metabolism [22]. More recently, several studies have characterized the temporal transcriptional program reflecting the cellular response to androgens, which led to the identification of novel androgen-regulated protein-coding genes [23-25].

In the present study, to identify androgen-responsive non-coding intronic RNAs, we used a custom-built spotted cDNA microarray enriched in intronic transcribed sequences and a prostate cancer cell line (LNCaP) cultured in the presence of a synthetic androgen. Using orientation-specific reverse transcription followed by PCR (RT-PCR) we determined the sense or antisense direction of a subset of intronic transcripts. We found that the levels of these intronic RNAs in androgen-treated and control cells correlate with the levels of message or to the alternative usage of exons in the corresponding protein-coding transcript. Additional approaches such as rapid amplification of the cDNA ends (RACE) and chromatin immunoprecipitation assays (ChIP) demonstrated that these intronic messages represent long and unspliced RNA transcripts that may be directly regulated by the androgen receptor (AR) *in vivo*. These findings indicate that transcription of long intronic ncRNAs may be controlled by mechanisms common to protein-coding transcripts, such as those involving hormonal control of gene-promoter activation. In addition, our results indicate that a fraction of this class of long unspliced intronic RNAs may have a role in post-transcriptional regulation of gene expression by modulating transcript stability and alternative splicing.

## Results and discussion

### Identification of intronic RNAs regulated by androgen

**Figure 1:**
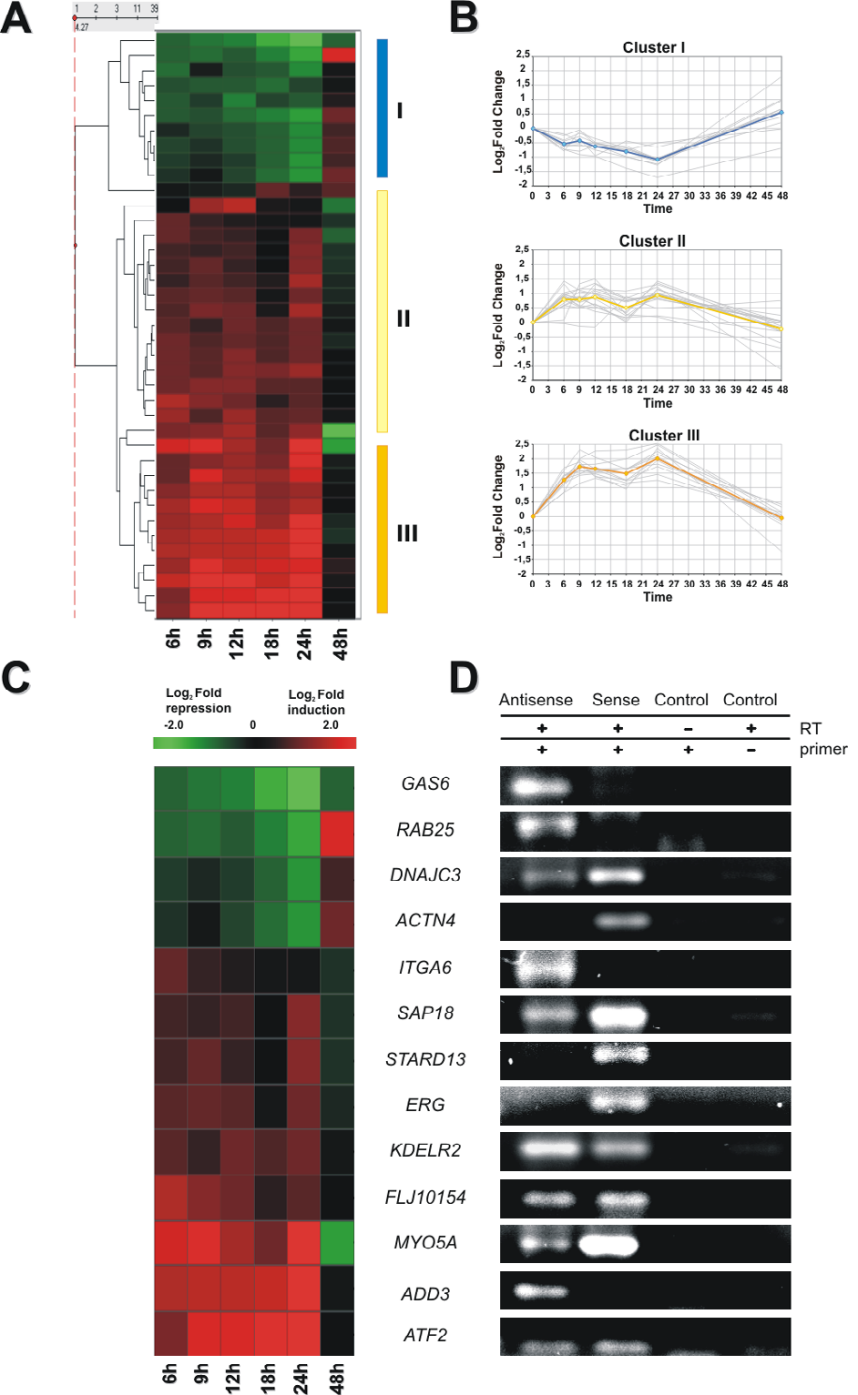
**Androgen-responsive intronic RNAs**. (A) Hierarchical clustering of 39 intronic transcripts significantly upregulated or downregulated after androgen exposure. (B) Clusters of intronic transcripts (I-III) with similar temporal expression patterns are indicated. Average fold change within each cluster in panel B is indicated by a colored line. (C) Microarray expression pattern of 13 selected androgen-regulated intronic messages selected for orientation-specific reverse-transcription PCR (OSRT-PCR). (D) Transcriptional orientation of 13 selected androgen-regulated intronic messages relative to the correspondent protein-coding mRNA determined by OSRT-PCR. Antisense (*GAS6*, *RAB25, ITGA6 *and *ADD3*), sense (*ACTN4*, *STARD3 *and *ERG*) or simultaneous antisense and sense (*DNAJC3, SAP18, KDELR2, FLJ10154, MYO5A *and *ATF2*) transcription (right panel; antisense, sense +RT, + primer) was observed. Controls to monitor the absence of contamination with genomic DNA (right panel; control - RT, + primer) or the absence of RNA self-priming (right panel; control + RT, - primer) are indicated. Names between panels C and D refer to protein-coding RefSeq mRNAs that map to the same loci and define intronic regions where active transcription was detected.

We used a spotted cDNA microarray platform enriched in intronic transcripts [16] to identify intronic RNAs and exonic protein-coding messages that had significant changes in transcription levels when hormone-responsive LNCaP prostate cancer cells were treated with the synthetic androgen R1881. Expression levels of *KLK3 *(*PSA*, prostate-specific antigen) [24] and *TMEPAI *[26], two genes previously shown to be upregulated by androgens in prostate epithelium, were determined by real-time PCR, confirming the effectiveness of androgens to induce responsive genes in our experimental conditions (see Additional file 1). Next, we used significance analysis of microarrays (SAM) [27] to identify transcripts that showed statistically significant changes in levels after exposure to androgen for periods of 6–48 hours. Only transcripts showing significant expression changes (≥ 1.5-fold change and false discovery rate < 5%) for at least three consecutive time points were selected for further analysis. We found 168 protein-coding exonic messages (see Additional file 2) and 39 intronic RNAs (Figure 1A) that had statistically different patterns of temporal expression in androgen-treated cells compared with untreated controls (the complete list of androgen-regulated exonic transcripts coding for proteins is shown in Additional file 3).

Androgen-regulated intronic RNAs could be grouped into three clusters based on their different temporal patterns of expression after androgen exposure (Figure 1B). Cluster I contained RNAs whose levels were 2–3-fold downregulated within 24 h after androgen exposure and were restored at 48 h. Clusters II and III included intronic RNAs that were upregulated by androgen within 24 h and later restored. Cluster II grouped RNAs that were 2-t3-fold increased, and cluster III grouped RNAs that were upregulated 3–6-fold. All of these intronic RNAs are unspliced messages, and apparently have no coding potential as determined by ESTScan analysis [28] (a complete list of androgen-regulated intronic RNAs is provided in Additional file 4).

Most mammalian snoRNAs [5] and a large fraction of microRNAs [29] are derived from intronic sequences of protein-coding and noncoding genes. To investigate the possibility that some of the androgen-regulated intronic transcripts might be primary transcripts that, after processing, could generate known small RNAs of those classes, we compared the sequence of the 39 androgen-regulated intronic RNAs with those of 346 snoRNAs [30] and 383 microRNAs [31]; no similarity was found.

We then investigated the enrichment of specific gene ontology (GO) categories for androgen-regulated exonic and intronic transcripts using BiNGO, a gene ontology comparison tool [32]. We postulated that intronic transcripts represent a new class of RNAs that might act as *cis-*regulatory factors [33]. Thus, for each androgen-regulated intronic transcript we used the GO annotation assigned to the corresponding protein-coding mRNA mapping to the same genomic locus. We found no significant enrichment of specific GO categories (*p *< 0.05) among the selected sets of exonic or intronic androgen-regulated transcripts when a correction for multiple testing was applied. However, we noted that 13 of the set of 39 androgen-regulated intronic transcripts belonged to the signal-transduction GO category (GO no. 0007165). Indeed, some of these loci have already been implicated in cellular events related to prostate cell growth and differentiation. For example, the protein encoded by *GAS6 *mRNA is a ligand of the Axl receptor tyrosine kinase, and the Gas6/Axl complex has been shown to exhibit mitogenic activity in human prostatic cancer cell lines by modulating the PI3K/AKT and MEK signal-transduction pathways [34]. Similarly, the transcription factor encoded by *ERG*, a member of the ETS family of central genes involved in integrating signals that regulate cell growth and differentiation, stress responses and tumorigenesis, has been previously identified as the most frequently overexpressed proto-oncogene in malignant prostate epithelial cells [35].

### Characterization of androgen-regulated intronic RNAs

Orientation-specific RT-PCR was performed (see Methods for details) on a selected set of androgen-regulated intronic RNAs (Figure 1C). Sense or antisense orientation of the intronic RNA relative to the corresponding protein-coding gene could be unambiguously assigned in most cases (13 of 17). For most of these (10 of 13) an intronic antisense RNA could be detected (Figure 1D). For six of these antisense RNAs (*DNAJC3*, *SAP18*, *KDELR2*, *FLJ10154*, *MYO5A *and *ATF2*) we detected an overlapping intronic sense message transcribed in the opposite strand. Four RNAs (*GAS6*, *RAB25*, *ITGA6 *and *ADD3*) were only detected in the antisense orientation, and three intronic messages (*ACTN4*, *STARD13 *and *ERG*) were only detected in the sense orientation (Figure 1D). The intronic sense transcripts of *ACTN4*, *STARD13 *and *ERG *did not show coding potential as determined by ESTScan. Without further experimentation, we could not determine if these intronic sense transcripts are novel non-coding exons present in alternative splicing forms, or if they represent ncRNAs originated from independent intronic transcriptional units located in the sense strand. However, the latter explanation concurs with sense intronic transcriptional units that have been observed elsewhere [9,36].

**Figure 2:**
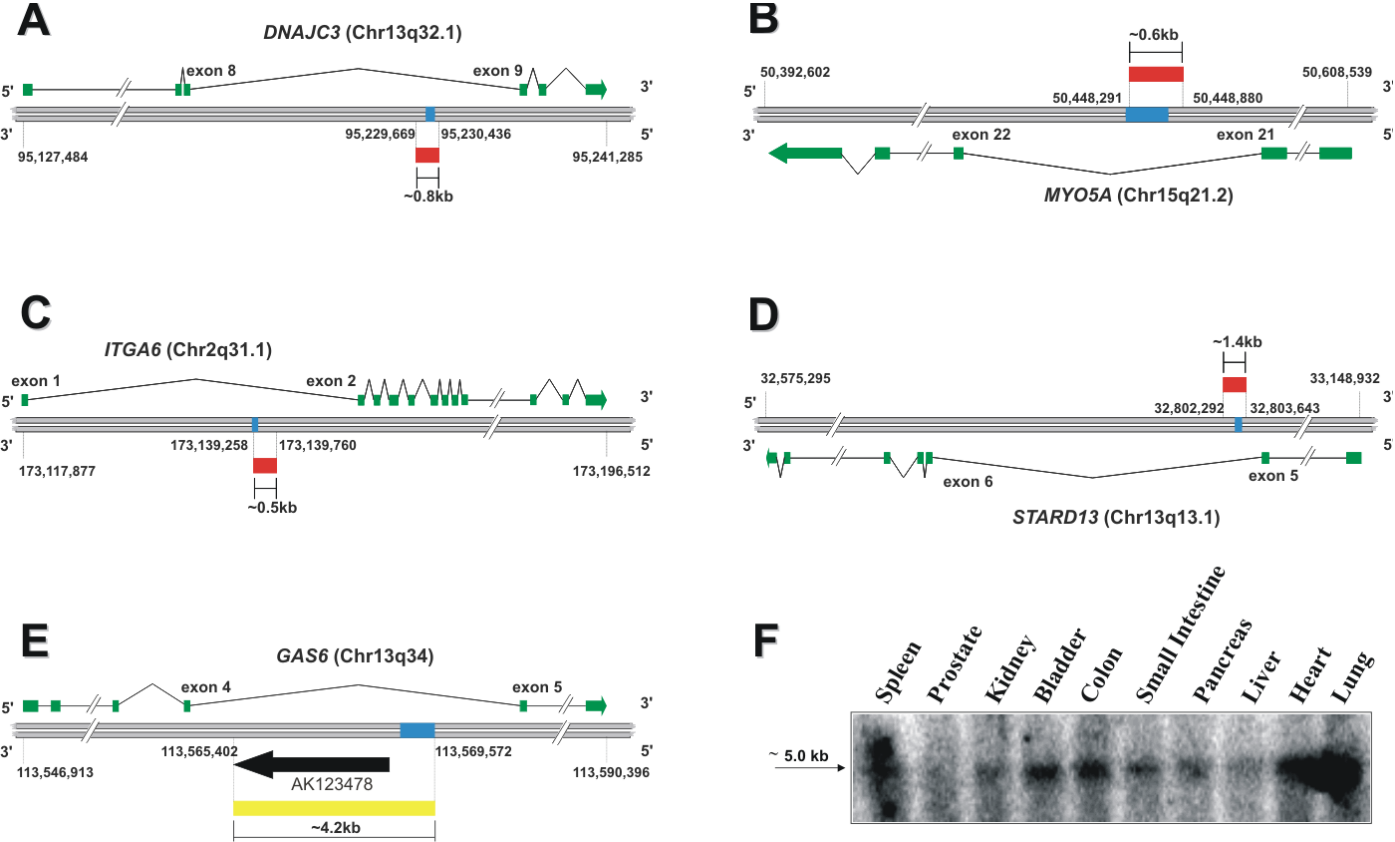
**Characterization of androgen-responsive non-coding intronic RNAs**. Selected intronic transcripts extended by RACE-PCR (A-D, red boxes) or gene-specific PCR (E, yellow box) are shown. Extended unspliced antisense intronic transcript fragments were mapped to the genomic sequence (A-E, gray lines) relative to the respective spliced protein-coding transcript expressed in the opposite strand (A-E, green arrows). Double-stranded cDNA clones spotted on the microarrays are represented as blue boxes (A-E). A previously described antisense unspliced intronic transcript mapping to *GAS6 *locus is shown (E, black arrow). Chromosome coordinates and the length of extended intronic transcript fragments are indicated. (F) Strand-specific multi-tissue northern blot using probes complementary to the antisense intronic transcript mapped in the *GAS6 *locus.

Next, RACE-PCR experiments with cDNA from prostate adenocarcinoma cells were performed for a selected set of intronic RNAs to obtain their full-length sequences. This selected set comprised intronic RNAs transcribed in the antisense (*GAS6 *and *ITGA6*), sense (*STARD3*) or both antisense and sense orientations (*MYO5A *and *DNAJC3*) as determined by orientation-specific RT-PCR. RACE experiments revealed that antisense intronic RNAs from *DNAJC3*,*MYO5A *and *ITGA6 *were long (0.5–0.8 kb), unspliced transcripts that mapped totally inside the intronic regions of the corresponding protein-coding gene (Figure 2A–C). An unspliced, totally intronic sense transcript from *STARD3 *of approximately 1.4 kb was obtained (Figure 2D). Further analysis of the coding-potential by ESTScan indicated that extended intronic *STARD3 *could generate a 74-residue peptide, but subsequent BLASTp searches [37] did not show any similarity of this small predicted product to known proteins in GenBank or to conserved protein domains at CDD [38]. We found in GenBank a 3.2-kb mRNA cloned from a brain cDNA library [GenBank: AK123478] that confirmed and extended the intronic antisense RNA mapping to the *GAS6 *locus. Next, we performed PCR with primers complementary to the cDNA probe mapping to an intron of *GAS6 *deposited in the microarray and to the AK123478 mRNA. We obtained a 4.2-kb amplicon, demonstrating that the *GAS6 *antisense intronic RNA transcribed in prostate cells was even longer (at least 4.2 kb) than the previously known RNA (Figure 2E, yellow bar).*In silico *analysis using ESTScan [28] failed to identify open reading frames for extended intronic sequences transcribed from *DNAJC3*, *ITGA6*, *MYO5A *and *GAS6 *loci.

A strand-specific northern blot confirmed the expression of a long (~5 kb) *GAS6 *antisense transcript in various human tissues (Figure 2F). The observation that androgen-regulated intronic RNAs could be detected in tissues that are not particularly responsive to this hormone (Figure 2, panel F) suggests that regulation of the steady-state levels of these transcripts may also proceed through an androgen-independent pathway.

### Effect of androgen in the levels of intronic RNAs and corresponding protein-coding transcripts

Of the androgen-regulated transcripts identified in our analysis, only *KDELR2 *and *ADD3 *showed detectable changes in expression levels of both intronic and exonic transcripts. The intronic *ADD3 *transcript increased 4.9-fold (log_2 _ratio = 2.3) in androgen-treated cells, whereas the corresponding protein-coding transcript decreased 3.3-fold (log_2 _ratio = -1.7) in the same experiment. Intronic *KDELR2 *transcript levels increased 2-fold (log_2 _ratio = 1), and the corresponding exonic transcript increased 2.3-fold (log2 ratio = 1.2) in cells exposed to androgen relative to control cells (see Additional files 3 and 4). These results indicate that for a given intronic-exonic pair transcribed in the same locus, the intronic message may be directly or inversely modulated with respect to the exonic message after androgen treatment.

**Figure 3:**
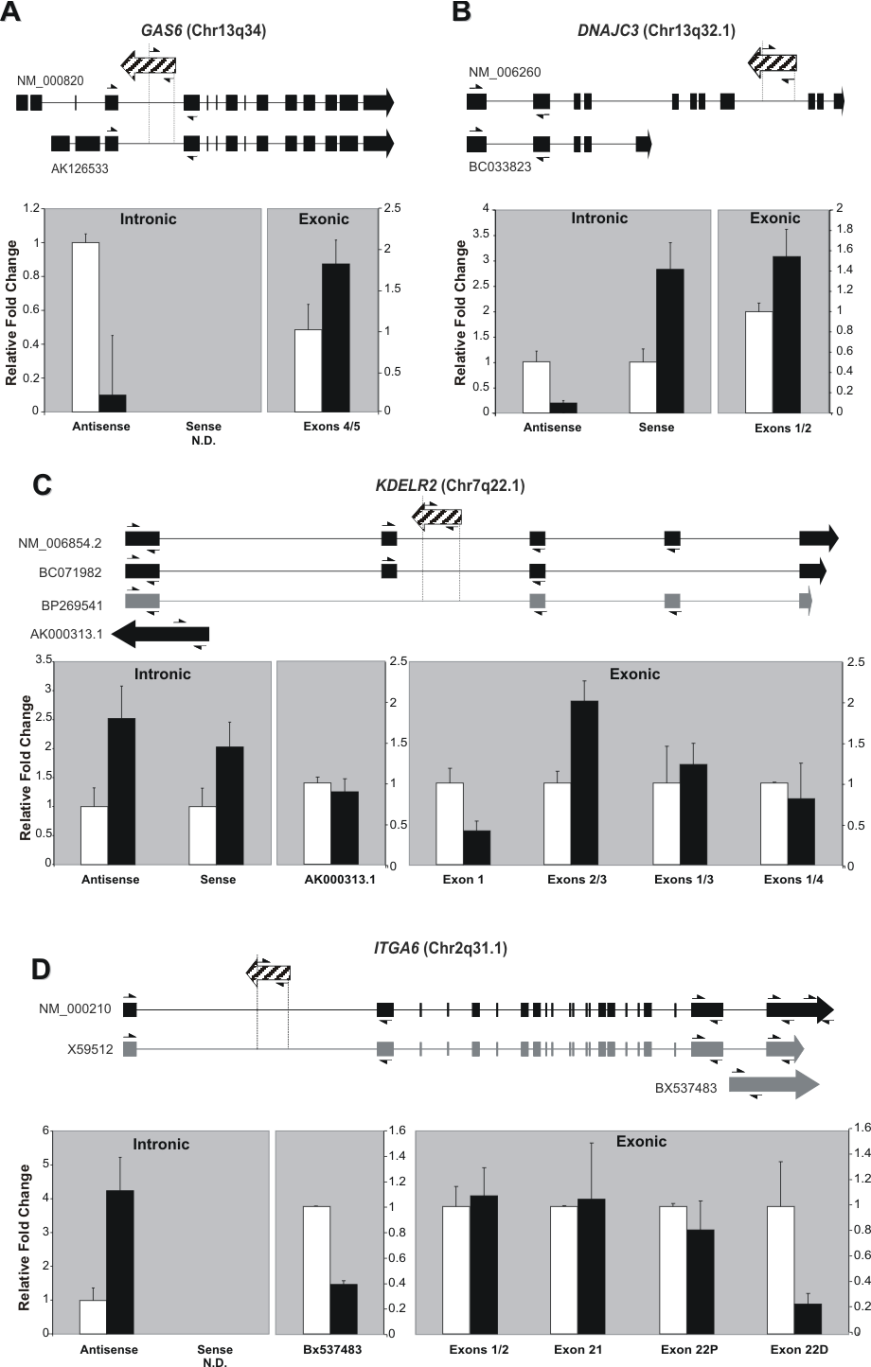
**Antisense intronic transcription may regulate the levels and the post-transcriptional processing of sense protein-coding mRNAs**. (A-D) Expression levels of sense, antisense intronic transcripts ("intronic" panels) or specific exons of protein-coding messages ("exonic" panels) from *GAS6 *(A), *DNAJC3 *(B), *KDELR2 *(C) or *ITGA6 *(D) loci were measured in androgen-treated (black bars) or control cells (white bars). Full-length curator-confirmed mRNAs mapping to each loci are shown as black arrows; non-curator-confirmed mRNAs and ESTs are shown by gray arrows; androgen-regulated intronic unspliced transcripts are shown by hashed arrows. Relative fold change (Y axis) indicates the increase or decrease in transcript levels after androgen-treatment relative to their levels in untreated control cells. Error bars refer to standard deviations relative to mean fold-change values.

Orientation-specific reverse transcription, followed by quantitative real-time PCR using RNA from treated and control untreated cells, was used to confirm the change in levels of intronic transcripts after androgen exposure. We tested two intronic RNAs that were detected only in the antisense orientation (*GAS6 *and *ITGA6*), and two intronic messages in which both antisense and sense transcription were measured (*DNAJC3 *and *KDELR2*) (Figure 3A–D, "intronic" panels). In each case, we quantified in parallel the expression levels of exons of the corresponding protein-coding gene (Figure 3A–D, "exonic" panels). We confirmed by quantitative PCR a 10-fold decrease of the intronic antisense transcript to *GAS6 *after androgen stimulation (Fig 3A, intronic panel). Interestingly, protein-coding messages for *GAS6 *that contain exons 4 and 5 were upregulated (1.8-fold) under identical experimental conditions (Figure 3A, exonic panel). We observed that the levels of sense and antisense intronic RNAs at the *DNAJC3 *locus were modulated in opposite directions after androgen treatment (Figure 3B, intronic panel). The upregulation observed for the sense intronic RNA from *DNAJC3 *after androgen exposure was in agreement with the results from the microarray experiments (Figure 3B, intronic panel), and was also observed for the protein-coding *DNAJC3 *transcript (Figure 3B, exonic panel). Modification in the relative levels of intronic RNAs from *KDELR2 *and *ITGA6 *after androgen treatment was confirmed (Figure 3C and 3D, intronic panels). For *KDELR2*, both antisense and sense intronic RNA levels were upregulated by androgen (2.5-fold and 2.0-fold increase, respectively; Figure 3C, intronic panel). Likewise, the antisense intronic RNA at the *ITGA6 *locus was 4.2-fold increased in the presence of androgen (Figure 3D, intronic panel). However, in these cases we could not detect notable changes in the relative levels of the respective protein-coding transcripts when primers designed to measure constitutive exons were used (Figure 3C and 3D, exonic panels).

To accumulate further evidence of functional involvement of antisense intronic non-coding transcripts on the splicing mechanism [19,33], we investigated the existence of correlation between their relative levels and the levels of the corresponding protein-coding mRNAs, using primers designed to probe regulated exons, that is, exons that are absent in at least one documented transcript isoform. At the *KDELR2 *locus, we found that exons 2 and 3 were upregulated (2-fold) after androgen treatment (Figure 3C, exonic panel). Notably, exon 2 was absent in one *KDELR2 *transcript isoform [GenBank: BP269541]. Therefore, it is conceivable that the observed increase in the level of intronic antisense RNA for *KDELR2 *after androgen treatment may induce increased retention of exon 2. We could not detect any notable androgen-driven change in the levels of transcripts containing exons 1 and 3 or exons 1 and 4 (Figure 3C, exonic panel). Notwithstanding, we observed that exon 1 for *KDELR2 *was downregulated 2.4-fold after androgen treatment when a pair of primers designed to probe only this exon was used (Figure 3C, exonic panel). This result can only be accounted for by the accumulation after androgen treatment of an as yet uncharacterized alternative *KDELR2 *transcript with a shorter 3' end.

At the *ITGA6 *locus we found that the levels of a protein-coding transcript isoform containing a longer terminal exon (exon 22D) were downregulated 4.2-fold after androgen treatment (Figure 3D, exonic panel), and thus inversely correlated with the 4.2-fold increase observed in the antisense intronic RNA under the same conditions (Figure 3D, intronic panel). However, it was not possible to determine whether this result was actually due to downregulation of the *ITGA6 *mRNA isoform containing exon 22D, as we also detected downregulation of another intronic transcript naturally transcribed in the *ITGA6 *locus [GenBank: BX537483] that spans the 3' terminal exon of the gene (Figure 3D).

Non-coding RNAs have been shown to affect splicing of protein-coding genes [19,39]. *HBII-52*, a snoRNA was found to regulate alternative splicing of the serotonin receptor gene *5-HT2CRA *in *trans*, by binding to a silencing element in exon Vb of *5-HT2CRA *[39]. Lack of *HBII-52 *expression causes defective *5-HT2CRA *pre-mRNA processing and probably accounts for the behavioral problems observed in patients with congenital Prader-Willi syndrome [39]. A previous study on the *FAS *locus has identified a partially intronic antisense RNA that is naturally transcribed from the first intron of the gene, which was named *Saf *[19]. *In vitro *overexpression of *Saf *induced skipping of several 3' exons of *Fas *mRNA, resulting in a Fas protein that was no longer able to anchor to the cell membrane or to induce Fas-mediated apoptosis [19]. Our results identify novel intronic antisense RNAs that might be involved in regulatory mechanisms of alternative splicing of protein-coding genes, in analogy to those observed for *HBII-52 *and *Fas/Saf*.

### Increased binding of the androgen receptor to an androgen response element motif upstream of *asMYO5A*

Androgen-mediated transcriptional regulation involves direct hormone interaction with the AR protein and the translocation of this complex to the nucleus, promoting specific interactions with DNA (i.e. androgen response element; ARE) motifs located near or within the target sequences [40]. To support a mechanism of direct transcriptional control of androgen-regulated intronic RNAs, we used a bioinformatics approach [24] to look for putative ARE motifs located upstream of the identified androgen-regulated intronic RNAs. At least one putative ARE motif was identified for 24 androgen-regulated intronic transcripts (a complete list of identified putative ARE motifs is presented in Additional file 5).

**Figure 4:**
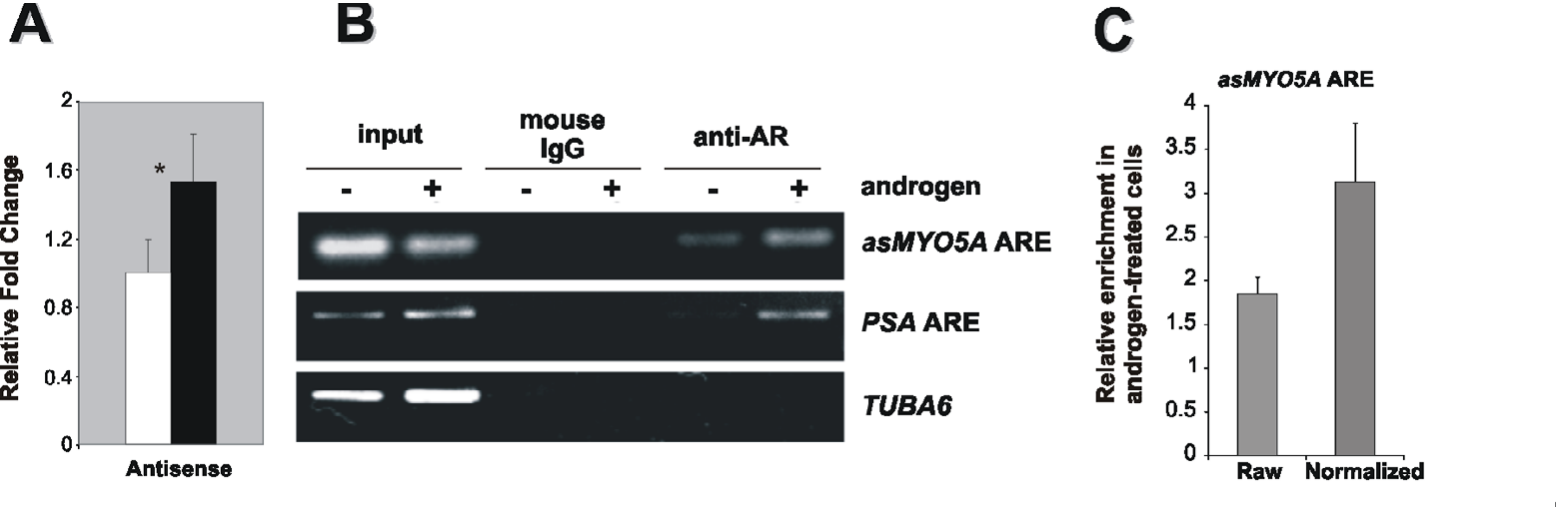
**Increased levels of antisense intronic MYO5A transcript correlates to binding of androgen receptor to a putative upstream androgen response element**. (A) Expression level of antisense intronic transcript of *MYO5A *was measured in androgen-treated (black bars) or untreated control (white bars) cells. Relative fold change (Y axis) shows the increase or decrease in transcript levels after androgen treatment relative to their levels in untreated control cells. Error bars refer to standard deviations relative to mean fold-change values. Differential expression of antisense *MYO5A *transcripts was statistically significant (*p *< 0.05) as indicated by asterisks. (B) Cellular lysates from androgen-treated (+) or control cells (-) were used in chromatin immunoprecipitation (ChIP) assays in the presence of an antibody reactive with the androgen receptor protein (anti-AR). PCR reactions were used for detection of immunoprecipitated DNA containing an androgen response element (ARE) located upstream to the *MYO5A *antisense intronic transcript (*asMYO5A *ARE) in androgen-treated (+) or untreated control cells (-). Prostate-specific antigen ARE (*PSA *ARE), or a non-related genomic sequence upstream of the tubulin α6 gene (*TUBA6*) were used as positive and negative PCR controls, respectively. An unrelated antibody (mouse IgG) was used as a control for nonspecific immunoprecipitation. Equal volumes of lysates from androgen-treated or control cells (1:100 of total lysate) were treated in parallel, and used as loading controls of input DNA for each ChIP assay (input). (C) Enrichment ratios of *asMYO5A *ARE bound to AR in androgen-treated cells relative to control cells. Enrichment ratios (Raw) represent mean ratio values calculated from the integrated band intensities obtained at two different PCR cycle numbers (25 and 30 cycles), in reactions similar to those of Figure 4B, as described in Methods. Band intensities from input DNA were used to obtain normalized enrichment ratios (normalized). Error bars show the standard deviation of mean ratios.

ChIP assays were performed to verify functional binding of AR to a putative regulatory ARE motif located upstream of the intronic antisense RNA mapping to the *MYO5 *locus. The assays revealed that after androgen treatment there was a 2–3-fold increase in *asMYO5A *ARE motifs bound to ARs (Figure 4B and 4C, *asMYO5A *ARE). The *asMYO5A *ARE motif is located at a 5' genomic region 812 bp upstream of the region where the antisense *MYO5A *intronic transcript maps. Binding of AR to a previously described functional ARE that regulates transcription of the *PSA *gene was tested in parallel as a positive control for the ChIP assay (Figure 4B, *PSA *ARE). A genomic region upstream from *TUBA6*, a gene that does not contain putative ARE motifs in its promoter region, was used as a negative control for non-specific immunoprecipitation of DNA fragments (Figure 4B, *TUBA6*).

The presence of a weak band for *asMYO5A *ARE in control cell lysates immunoprecipitated with anti-AR (Figure 4B, *asMYO5A *ARE), indicating that binding of AR to *asMYO5A *ARE may occur to some extent in untreated cells. This may be explained by the presence of trace amounts of androgen remaining in charcoal-stripped fetal calf serum added to the cell-culture medium. An alternative explanation for the background signal of *asMYO5A ARE *measured in control cells may be the existence of small amounts of AR constitutively bound to the chromatin, as described for another transcription factor, cyclic AMP-responsive element-binding protein (CREB) [41].

The increased binding of AR to the *asMYO5A *ARE motif is in agreement with the androgen-induced increased expression of the antisense intronic *MYO5A *RNA (*asMYO5A*) detected by microarray (Figure 1), and confirmed by orientation-specific reverse transcription followed by real-time PCR (Figure 4A). *MYO5A *encodes an actin-based processive motor involved in intracellular trafficking and exocytosis [42]. Tissue-specific Myo5a isoforms are originated from alternative exon usage of the primary transcript, which in turn is thought to modulate the protein specificity for different cargoes within the cell [43]. It is conceivable that the increase in *asMYO5A *levels in prostate cells in response to androgen affects the relative levels of the alternatively spliced forms of *MYO5A*, thus modulating protein activity or specificity. Together, these results suggest that functional binding of androgen-AR activated complexes to regulatory ARE regions is required to modulate the levels of intronic antisense RNAs after androgen treatment of prostate cells.

## Conclusion

Although there is still no conclusive evidence about the molecular mechanisms involved in response to androgen, we postulate that intronic transcripts exert their regulatory functions by acting in *cis *through Watson-Crick base-pairing to complementary sense pre-mRNA [33]. The results presented here corroborate the notion that at least a fraction of these intronic transcripts may directly affect the levels and/or splicing of the protein-coding transcript originating in the opposite strand, and concur with previous reports in the literature [44,45].

It is not yet clear if the intronic transcripts identified in this work exert their potential regulatory functions as long (0.5–5 kb) RNAs, or alternatively, as shorter RNAs resulting from processing of the primary transcript. Processing of these androgen-regulated ncRNAs into shorter RNAs of defined sizes would also raise the possibility that some may modulate transcriptional or post-transcriptional events in messages from different loci, in a manner similar to microRNAs [4]. The identification of possible short RNAs modulated by androgen will require further work, such as 15% denaturing polyacrylamide gel electrophoresis or northern blots of prostate cells exposed to androgen in time-course experiments to document the accumulation of the long intronic RNAs, the time course of their conversion to short RNAs, and the accumulation of possible short RNA intermediates.

Previous reports have shown that a large number of transcription-factor binding sites (TFBSs) for well-characterized transcription factors such as Sp1, c-Myc, p53 and Creb lie upstream to transcriptionally active intronic regions [41,46], some of which generate transcripts that are oriented antisense relative to the known gene [46]. Notably, Cawley *et al *[46] found that comparable fractions of protein-coding genes and ncRNAs having an experimentally determined upstream TFBS region were regulated after exposure to retinoic acid. The results presented here provide further evidence that ncRNAs transcribed from intronic segments of the human genome may be regulated by physiological signals that commonly act on protein coding-transcripts such as hormones. Similar results were observed in mice when the effect of lipopolysaccharide stimulation on the levels of several long ncRNAs was measured [47]. Together, these results strongly suggest that at least in mammalian cells intronic ncRNAs are transcribed in a regulated fashion and therefore must exert physiological roles, which corroborates the existence of a yet underappreciated complex RNA-based regulatory system involving non-coding messages in higher organisms [2,3,48].

## Methods

### Cell culture and RNA isolation

The experimental design was based on recent published work [24]. In summary, prostate carcinoma cell lines LNCaP, DU145 and PC3 were obtained from the American Type Culture Collection and maintained using the suggested medium supplemented with 10% (v/v) fetal calf serum (FCS), 3 mM L-glutamine, 100 μg/ml streptomycin and 100 U/ml penicillin. LNCaP cells were grown in RPMI 1640, and DU145 and PC3 cells were cultured in DMEM. For the androgen-response experiments, LNCaP cells were cultured for 24 hours in androgen-deprived medium (RPMI 1640) with 5% (w/v) charcoal-stripped fetal calf serum (FCS; Invitrogen, Carlsbad, CA, USA). After 24 hours, cells were placed in fresh RPMI 1640 medium, with 10% (w/v) charcoal-treated FCS, and either 1 nM of the synthetic androgen R1881 (Perkin Elmer, Wellesley, MA, USA) or equivalent volume of vehicle (ethanol) was added. Cells were harvested after 0, 6, 9, 12, 18, 24, and 48 hours for RNA isolation. Two independent cultures of androgen-treated cells and of control cells (independent biological replicates), were used for RNA isolation.

In parallel, cells were harvested after 4 hours of hormone treatment for isolation of DNA that was subsequently used in ChIP assays, as described below. Total RNA was purified from experimental and control cells by caesium chloride cushion [49] after cell lysis (4 M guanidine isothiocyanate; 0.1 M β-mercaptoethanol; 25 mM sodium citrate pH 7.0). Total RNA (100 μg sample) was treated with RNase-free DNase (RNeasy kit; Qiagen, Hilden, Germany) for 15 min in the medium recommended by the manufacturer to minimize genomic DNA contaminants, and kept at -80°C until use.

### Microarray experiments

The microarray platform used in this study was constructed using PCR-amplified cDNA fragments derived from ORESTES clones [50], and spotted onto Type 7 STAR glass slides (GE Healthcare, Piscataway, NJ, USA) as previously described [16]. This consists of a 9216-element microarray (3355 unique cDNA probes spotted in duplicate, plus positive and negative controls) enriched in probes representing intronic (822 probes) and intergenic (241 probes) ncRNAs. For each time point, 15 μg of DNAse-treated total RNA from androgen-treated or untreated control LNCaP cells were used to generate fluorescent targets by incorporation of Cy5-dUTP in reverse transcription reactions using the CyScribe First strand cDNA labeling kit (GE Healthcare) and the protocol recommended by the manufacturer. Fluorescent targets labeled with Cy3-dUTP were generated in parallel from a reference RNA pool consisting of equimolar quantities of DNAse-treated total RNA from LNCaP, DU145 and PC3 cells.

For each independent biological replicate hybridization, fluorescently labeled targets from each experimental time point and the reference pool were combined and hybridized on an automated slide processor (GE Healthcare) using protocols recommended by the manufacturer. For each time point, two independent biological replicate hybridizations were performed. Each hybridization used one RNA, isolated from a different biological replicate of androgen-treated or untreated control cells. Processed slides were scanned in a microarray scanner (GenePix 4000 B; Molecular Devices, Sunnyvale, CA, USA). More detailed information about microarray experiments and analysis is provided as supplementary material (see Additional file 6) following MIAME guidelines [51].

### Data acquisition and statistical analysis

Background-subtracted artifact-removed median intensities of both Cy3 and Cy5 emissions were extracted for each spot from raw images using image analysis software (ArrayVision version 8.0; GE Healthcare). Only spots with signal intensity above the average plus 3 SD from a set of negative controls (plant and bacterial DNA) were used in subsequent analyses. As each microarray contains two replicates of each spotted cDNA, a total of four replicate measurements was obtained for each spot at each time point. We evaluated the average coefficient of variation between replicates of expression ratios between treated and control cells calculated in two different ways. For each time point, expression ratios between treated and control cells were reconstructed either from (i) ratios relative to the reference pool or (ii) Cy5 intensities obtained from androgen-treated and control cells using a one-color approach [52]. A smaller average coefficient of variation between replicates was obtained with the latter approach, and therefore we opted to use only the Cy5-labeled sample measurements to calculate the treatment:control sample ratios. We used the mean intensity signal (40% trimmed) of each dataset for normalization between experiments. Raw and normalized microarray intensities were deposited in the Gene Expression Omnibus (GEO) database [53] (accession number GSE5345).

Transcripts with statistically significant changes in expression in response to androgen stimulation were identified using the statistical analysis of microarrays (SAM) method [27]. Only transcripts showing significant expression changes in at least three consecutive time points were selected for further analysis, using as parameters the two-class response (paired data), 1000 permutations, K-nearest neighbors imputer, fold change ≥ 1.5 and FDR < 5%. Expression profiles of SAM-selected transcripts were grouped using hierarchical clustering (UPGMA with Euclidean distance) and visualized using software (Spotfire Decision Site; Spotfire, Somerville, MA, USA).

### Orientation-specific RT-PCR and orientation-specific real-time quantitative RT-PCR

Orientation-specific RT-PCR was performed as previously described [16]. Briefly, 4 μg of DNase-treated total RNA from androgen-treated or control LNCaP cells were used to generate strand-specific cDNA. RNA samples were selected from experimental time points that showed higher fold change relative to control cells as identified in microarray hybridizations. The same RNA samples were used in microarray hybridizations and real-time PCR reactions. To determine the levels of sense and antisense intronic transcripts in androgen-treated and control cells, we performed one orientation-specific reverse transcription reaction for each strand using a single RNA sample, and gene specific (forward or reverse) primers. RNA samples plus 0.9 μM of specific primer for either antisense or sense strand of each intronic segment were heated to 75°C for 10 minutes to minimize artifacts derived from the formation of secondary structure. Reverse transcription was performed at 50°C for 60 min in a 50-μl volume, using 200 U of reverse transcriptase (SuperscriptII; Invitrogen). Controls for the absence of self-priming were obtained by performing reverse transcription in the absence of primers, and controls for the absence of genomic DNA contamination were obtained by incubation with primers in the absence of reverse transcriptase enzyme. A reaction containing a primer complementary to the coding region of *TUBA6 *(reverse primer: AGTGCCAGTGCGAACTTCATC) was carried out in parallel. Reverse transcriptase was denatured for 30 min at 95°C. One pair of internal primers for PCR amplification of either the tubulin gene or the desired target intronic segment under study was added. For each intronic transcript, cDNAs with sense or antisense orientation relative to the corresponding gene were analyzed by three replicate measurements in quantitative real-time PCR reactions. For exonic transcripts, cDNA was obtained by reverse transcription of RNA samples using oligo-dT primers, and the levels of each message was also determined by three replicate measurements in real-time PCR reactions with gene specific primers. All real-time PCR reactions were carried out in a volume of 20 μl for 40 cycles, according to the manufacturer's instructions for the reagent (Sybr-Green PCR core reagent; Applied Biosystems, Foster City, CA, USA) using an automated sequence analyser (GenAmp 5700; Applied Biosystems). For quantitative results, the levels of each transcript detected by real-time quantitative PCR was normalized to the level of tubulin, and represented as fold change using the ΔCt method [54]. Quantitative results for a transcript in androgen-treated cells were expressed as relative fold change with respect to the value of the target transcript in the control (ethanol vehicle), which was set at unity.

### Rapid amplification of cDNA ends-PCR and northern blot

For RACE-PCR we used a commercial cDNA library prepared from poly(A)+ RNA isolated from a human prostatic adenocarcinoma cell line (Human XG marathon-ready cDNA, catalog no. 7498-1; BD Biosciences, Palo Alto, CA, USA). Two rounds of PCR reactions were performed for each intronic transcript using two pairs of gene-specific primers in nested PCR reactions. To obtain a long (~5 kb) transcript corresponding to *asGAS6*, a gene-specific PCR was performed using a forward primer complementary to the partial *asGAS6 *cDNA sequence deposited in the microarray and a reverse primer complementary to the antisense transcript deposited in [GenBank: AK123478]. The PCR product was sequenced to confirm the identity. To confirm the size and orientation of the full-length *asGAS6 *obtained by PCR, a northern blot was performed using a commercial membrane containing 2 μg poly(A)+ RNA from 10 human tissues (First Choice Human Blot 4; Ambion, Austin, TX, USA), using a probe complementary to the antisense strand of *GAS6 *intronic transcript spotted onto the microarray. Sense α-[^32^P]-CTP-labeled RNA probes were obtained by *in vitro *transcription using T7 RNA polymerase (Riboprobe; Promega, Madison, WI, USA). Hybridization and wash procedures were carried out as described in the human blot manufacturer's protocol (Ambion). The probed blot was processed in a phosphorimager (Storm; GE Healthcare).

### Putative identification of androgen-responsive elements and chromatin immunoprecipitation assay

Using a bioinformatics approach, we searched for regions with similarity to the palindromic ARE consensus AGAACAnnnTGTTCT [40], obtained from the TRANSFAC database of eukaryotic *cis*-acting regulatory DNA elements [55]. The search was conducted within a 3-kb region of genomic sequence, upstream of the putative transcriptional start sites of the 39 androgen-regulated intronic transcripts identified in the microarray analysis. When available, RACE-extended fragments were used. ChIP assays were performed as described in the literature [56-58], and specific PCR primers were designed to detect binding of the androgen receptor to the putative ARE promoter sequence of *asMYO5A*, identified by *in silico *analysis. A chromatin immunoprecipitation assay kit (catalog no. 17–295, Millipore, Billerica, MA, USA) was used, with the protocols recommended by the manufacturer. Briefly, LNCaP cells treated with androgen or vehicle as described above were fixed with 1% formaldehyde for 10 min at room temperature and lysed with sodium dodecyl sulfate lysis buffer. Cell lysates were sonicated to shear the DNA between 200 and 1000 bp using a sonic dismembrator (Model 500; Fischer Scientific, Waltham, MA, USA). Aliquots of the lysate (100 μL). containing approximately 2 × 10^6 ^cells were diluted in 900 μL dilution buffer, and 10 μL from each aliquot (1% of total lysate) were separated and used subsequently as loading controls for input DNA. Parallel chromatin immunoprecipitation reactions were performed with either 3 μg polyclonal antibody anti-AR (H-280; Santa Cruz Technologies, Santa Cruz, CA, USA) or 1 μg normal mouse IgG (negative control). Immunoprecipitated chromatin fractions and respective loading control fractions were treated for cross-link reversal, treated with RNase A and proteinase K, and DNA fragments were purified using Spin Columns. An aliquote (2 μl) from each eluted DNA was used as template in PCR reactions with the following primers flanking the putative *asMYO5A *ARE: forward, TTTGCTGGATAAGGATTCCCA; reverse, AGTTCCTTAAACTTTAGCTTAGAAAAAGGA. Similar reactions were performed with control primers designed to the known AREIII motif of the *PSA *(prostate-specific antigen) enhancer (positive control) (forward primer: GCTCAGCCTTTGTCTCTGATGA, reverse: TGCAAGATGATATCTCTCTCAGATCC) and to the *TUBA6 *non-related upstream genomic sequence (negative control) (forward primer: GACTACAGGTGTGCGCCATCAT, reverse: TGCCGTGTTCCAGGCAGTAG). For each DNA target, semi-quantitative PCR was performed by running parallel reactions with increasing numbers of PCR cycles (25, 30 or 40 cycles). Cycle runs of 25 and 30 PCR cycles resulted in non-saturated amplification of each DNA target in immunoprecipitated and input DNA fractions from androgen-treated or control cells (data not shown). Equal volumes from each PCR reaction were loaded and separated in a 3% agarose gel. The intensity of PCR-amplified bands was determined by gel densitometry (Image Master; GE Healthcare).

### Accession numbers

We have deposited in GenBank the following sequence data under the indicated accession numbers (in parentheses): antisense intronic *ITGA6 *[GenBank: DQ866759], antisense intronic *DNAJC3 *[GenBank: DQ866760], antisense intronic *MYO5A *[GenBank: DQ866761], intronic *STARD13 *[GenBank: DQ866762], antisense intronic *GAS6 *[GenBank: DQ866763], antisense intronic *RAB25 *[GenBank: DQ866751], antisense intronic *ADD3 *[GenBank: DQ866752], intronic *ACTN4 *[GenBank: DQ866753], intronic *SAP18 *[GenBank: DQ866754], intronic *ERG *[GenBank: DQ866755], intronic *KDELR2 *[GenBank: DQ866756], intronic FLJ10154 [GenBank: DQ866757], and intronic *ATF2 *[GenBank: DQ866758].

## Authors' contributions

RL, HIN, PPA, AMDS, SVA and EMR conceived the experiments. RL, HIN, PPA and FF performed the experiments. RL, HIN, PPA, SVA and EMR analyzed the data. RL and EMR wrote the manuscript. MCS, AMDS, SVA and EMR contributed reagents, materials or analysis tools. All authors read and approved the final manuscript.

## Supplementary Material

Additional File 1 Supplementary Figure 1. Induction of androgen-responsive genes in prostate cells. Click here for file

Additional File 2 Supplementary Figure 2. Temporal expression profile of androgen-responsive exonic RNAs. Click here for file

Additional File 3 Supplementary Table 1. Androgen-responsive exonic transcripts. Click here for file

Additional File 4 Supplementary Table 2. Androgen-responsive intronic transcripts. Click here for file

Additional File 5 Supplementary Figure 3. *In silico *identification of ARE motifs in upstream regions of androgen-regulated intronic RNAs. Click here for file

Additional File 6 MIAME file. Click here for file
