# Supplementary material for: Androgen responsive intronic non-coding RNAs
Source: BMC Biol. 2007 Jan 30;5:4. doi: 10.1186/1741-7007-5-4 (PMC1800835; doi:10.1186/1741-7007-5-4)

Supplementary Figure 1. **Induction of androgen-responsive genes in prostate cells.** Expression levels from androgen-regulated genes *KLK3* (*PSA*, Prostate Specific Antigen) and *TMEPAI* were determined by Real-Time PCR to confirm the effectiveness of androgen to induce responsive genes in LNCaP cells treated with synthetic androgen for 6 up to 48h. Mean fold-changes in *KLK3* (**Upper panel)** and *TMEPAI* (**Lower panel**) RNA levels at each time point relative to time 0 are shown for androgen-treated cells (black bars) and control untreated cells (white bars). Error bars reflect the number of standard deviations of the mean fold-change in each time point, calculated from two replicate measurements. A two-sample Student t-Test (two-tailed, unequal variance) was used to verify the significance of fold-change differences observed at each time point relative to time 0. For androgen-treated cells, all but one time points showed significant increase in *KLK3* transcript levels at $p < 0.05$ (except point 12h, $p < 0.06$). Conversely, for control cells no time point showed increase (or decrease) at the $p < 0.05$ significance level. The fold-change increase in *KLK3* or TMPAI transcript levels measured in androgen-treated cells relative to control cells, at each time point were all significant at $p < 0.05$.

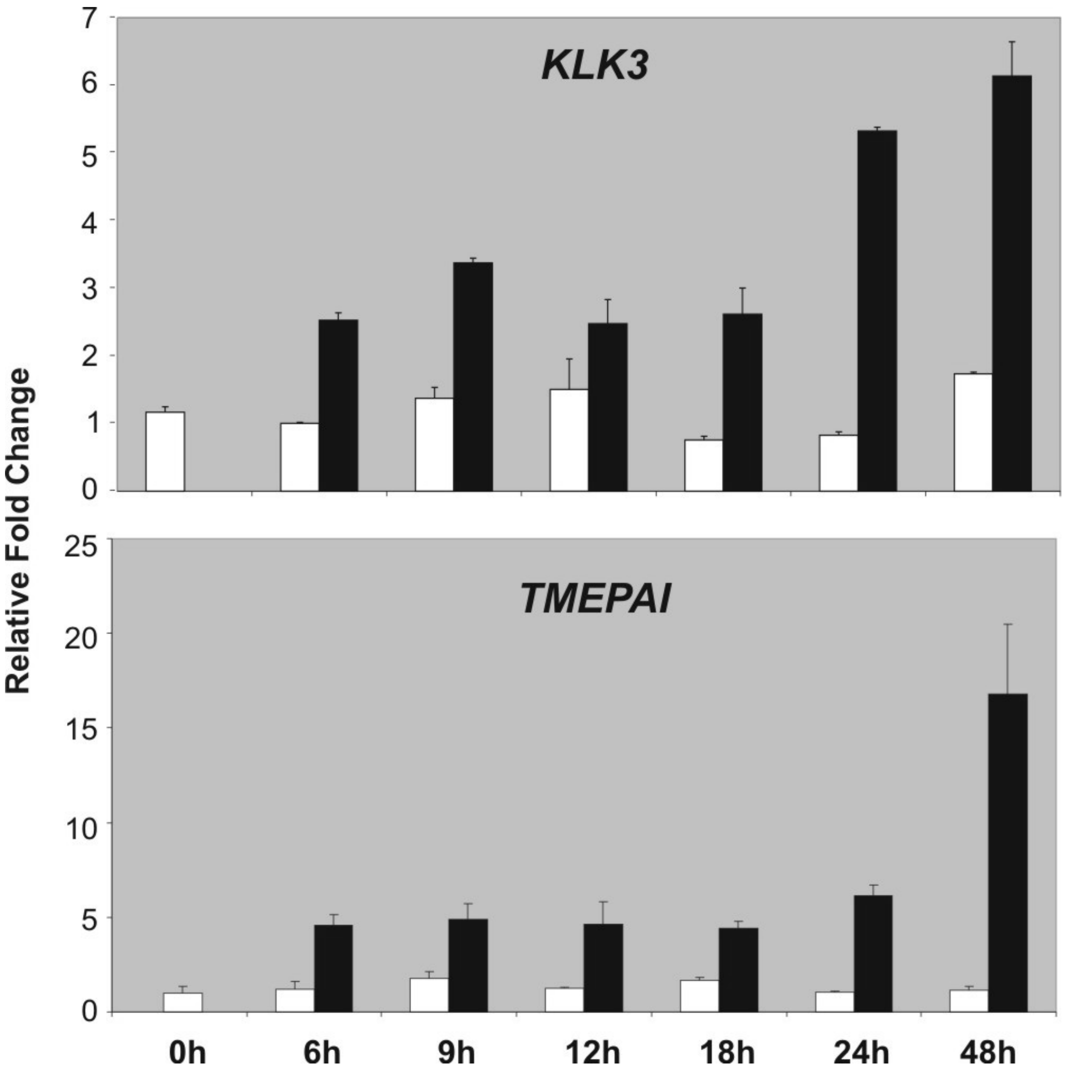

Supplement: Additional File 1 — Supplementary Figure 1. Induction of androgen-responsive genes in prostate cells. [file 1741-7007-5-4-S1.pdf]
