# Supplementary material for: Androgen responsive intronic non-coding RNAs
Source: BMC Biol. 2007 Jan 30;5:4. doi: 10.1186/1741-7007-5-4 (PMC1800835; doi:10.1186/1741-7007-5-4)

Supplementary Figure 2. **Temporal expression profile of androgen-responsive exonic RNAs.**

168 exonic transcripts significantly regulated (FDR < 5%) following androgen treatment of LNCaP cells relative to untreated control cells were selected as described in Methods section. Hierarchical clustering analysis of exonic transcripts significantly activated or repressed following androgen exposure is shown.

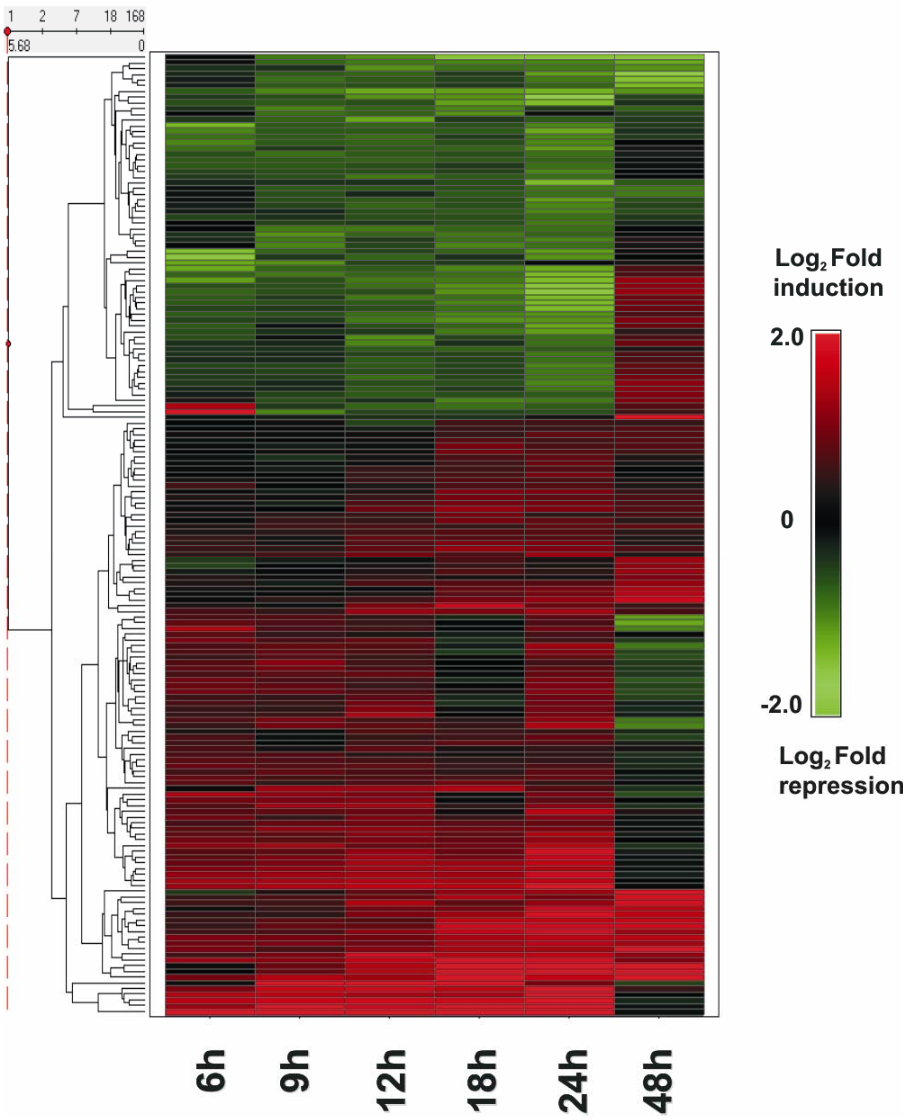

Supplement: Additional File 2 — Supplementary Figure 2. Temporal expression profile of androgen-responsive exonic RNAs. [file 1741-7007-5-4-S2.pdf]
