# Supplementary material for: Androgen responsive intronic non-coding RNAs
Source: BMC Biol. 2007 Jan 30;5:4. doi: 10.1186/1741-7007-5-4 (PMC1800835; doi:10.1186/1741-7007-5-4)
Supplement: Additional File 3 — Supplementary Table 1. Androgen-responsive exonic transcripts. [file 1741-7007-5-4-S3.pdf]

Supplementary Table 1 - Androgen-responsive exonic transcripts.

168 exonic transcripts with significant expression changes in at least 3 consecutive time points were identified by two-class SAM analysis and clusterized hierarchicaly as described in *Methods.*
Selection parameters were fold-change ≥ 1.5 and false discovery rate (FDR) < 5%. The fold-change (log2) column reflects the relative abundance of signficantly up- and downregulated protein-coding transcripts in LNCap cells treated with androgen for 24 hours, as compared to untreated control cells. Selected transcripts in the same order from the hierarchical clustering shown in Figure 1 A. N.A. - Not available

Spot ID	GenBank Accession (Spotted EST)	Mapping	Locus Name	Locus Annotation	GenBank Accession (from Annotation)	Androgen Effect	Fold-change (log2)	Exonic Hierarchical Clustering (order)
3299	BG876589	Exonic	UGT2B17	Homo sapiens UDP glycosyltransferase 2 family, polypeptide B17 (UGT2B17), mRNA	NM_001077	Downregulated	-2.54	1
3636	BF989967	Exonic	ARHGAP29	Homo sapiens Rho GTPase activating protein 29 (ARHGAP29), mRNA	NM_004815	Downregulated	-0.95	2
1225	BF805750	Exonic	COLEC12	Homo sapiens collectin sub-family member 12 (COLEC12), transcript variant I, mRNA	NM_130386	Downregulated	-0.92	3
1674	BF090400	Exonic	N.A.	Homo sapiens mRNA; cDNA DKFZp686K0548 (from clone DKFZp686K0548)	BX649128	Downregulated	-1.10	4
3668	AW995234	Exonic	C6orf55	Homo sapiens chromosome 6 open reading frame 55 (C6orf55), mRNA	NM_016485	Downregulated	-0.86	5
3321	BQ377372	Exonic	CPNE3	Homo sapiens copine III (CPNE3), mRNA	NM_003909	Downregulated	-0.70	6
3276	BQ366895	Exonic	SLAC2-B	Homo sapiens SLAC2-B (SLAC2-B), mRNA	NM_015065	Downregulated	-1.26	7
552	BF817791	Exonic	ADD3	Homo sapiens adducin 3 (gamma) (ADD3), transcript variant 1, mRNA	NM_016824	Downregulated	-1.70	8
565	BF945476	Exonic	MXI1	Homo sapiens MAX interactor 1 (MXI1), transcript variant 1, mRNA	NM_005962	Downregulated	-1.34	9
396	AW375604	Exonic	N.A.	Homo sapiens hypothetical protein (LOC387882), mRNA	NM_207376	Downregulated	-0.36	10
427	AW901818	Exonic	TMEM65	Homo sapiens transmembrane protein 65 (TMEM65), mRNA	NM_194291	Downregulated	-0.06	11
577	BF155848	Exonic	SYNE2	Homo sapiens spectrin repeat containing, nuclear envelope 2 (SYNE2), transcript variant 2, mRNA	NM_182910	Downregulated	-0.65	12
49	BE840007	Exonic	AMOTL1	Homo sapiens angiomotin like 1 (AMOTL1), mRNA	NM_130847	Downregulated	-0.99	13
245	BE165360	Exonic	ROCK1	Homo sapiens Rho-associated, coiled-coil containing protein kinase 1 (ROCK1), mRNA	NM_005406	Downregulated	-1.18	14

297	BF805983	Exonic	ADK	Homo sapiens adenosine kinase (ADK), transcript variant ADK-short, mRNA	NM_001123	Downregulated	-0.93	15
518	BE162922	Exonic	N.A.	Homo sapiens cDNA FLJ37408 fis, clone BRAMY2028491	AK094727	Downregulated	-0.99	16
642	BF883122	Exonic	CD63	Homo sapiens CD63 antigen (melanoma 1 antigen) (CD63), mRNA	NM_001780	Downregulated	-1.17	17
239	BE926612	Exonic	MNAT1	Homo sapiens menage a trois 1 (CAK assembly factor) (MNAT1), mRNA	NM_002431	Downregulated	-0.81	18
612	AW370601	Exonic	CNOT8	Homo sapiens CCR4-NOT transcription complex, subunit 8 (CNOT8), mRNA	NM_004779	Downregulated	-0.71	19
589	AW860776	Exonic	TERF1	Homo sapiens telomeric repeat binding factor (NIMA-interacting) 1 (TERF1), transcript variant 2, mRNA	NM_003218	Downregulated	-0.79	20
645	BF328037	Exonic	UBAP2L	Homo sapiens NICE-4 protein (NICE-4), mRNA	NM_014847	Downregulated	-0.92	21
1610	BG009879	Exonic	N.A.	Homo sapiens mRNA; cDNA DKFZp779K2051 (from clone DKFZp779K2051)	BX537506	Downregulated	-0.79	22
695	BE768777	Exonic	ST7	Homo sapiens suppression of tumorigenicity 7 (ST7), transcript variant a, mRNA	NM_018412	Downregulated	-1.40	23
2546	BF087381	Exonic	TP53	Homo sapiens tumor protein p53 (Li-Fraumeni syndrome) (TP53), mRNA	NM_000546	Downregulated	-0.92	24
1346	AW880649	Exonic	TMSB4X	Homo sapiens thymosin-like 3 (TMSL3), mRNA	NM_183049	Downregulated	-0.77	25
1410	BG012653	Exonic	RALB	Homo sapiens v-ral simian leukemia viral oncogene homolog B (ras related; GTP binding protein) (RALB), mRNA	NM_002881	Downregulated	-1.13	26
2569	AW937823	Exonic	MYC	Homo sapiens v-myc myelocytomatosis viral oncogene homolog (avian) (MYC), mRNA	NM_002467	Downregulated	-0.95	27
1031	BE074224	Exonic	CD151	Homo sapiens CD151 antigen (CD151), transcript variant 2, mRNA	NM_139030	Downregulated	-0.95	28
613	BF951773	Exonic	RAN	Homo sapiens RAN, member RAS oncogene family (RAN), mRNA	NM_006325	Downregulated	-0.80	29
791	BE815309	Exonic	ARRDC4	Homo sapiens arrestin domain containing 4 (ARRDC4), mRNA	NM_183376	Downregulated	-0.88	30
392	BF809098	Exonic	ZFP36L2	Homo sapiens zinc finger protein 36, C3H type-like 2 (ZFP36L2), mRNA	NM_006887	Downregulated	-0.82	31
1049	BE168033	Exonic	NFKB1	Homo sapiens nuclear factor of kappa light polypeptide gene enhancer in B-cells 1 (p105) (NFKB1), mRNA	NM_003998	Downregulated	-0.73	32

479	AW859979	Exonic	N.A.	Homo sapiens cDNA FLJ38048 fis, clone CTONG2014264	AK095367	Downregulated	-0.97	33
475	BF761020	Exonic	PALM2-AKAP2	Homo sapiens PALM2-AKAP2 protein (PALM2-AKAP2), transcript variant 1, mRNA	NM_007203	Downregulated	-0.77	34
214	BF754489	Exonic	FAU	Homo sapiens Finkel-Biskis-Reilly murine sarcoma virus (FBR-MuSV) ubiquitously expressed (fox derived); ribosomal protein S30 (FAU), mRNA	NM_001997	Downregulated	-1.12	35
231	BE165509	Exonic	DEFB1	Homo sapiens defensin, beta 1 (DEFB1), mRNA.	NM_005218	Downregulated	-1.08	36
3	AW994959	Exonic	ROD1	Homo sapiens ROD1 regulator of differentiation 1 (S. pombe) (ROD1), mRNA	NM_005156	Upregulated	0.08	37
273	AW373943	Exonic	CAPNS1	Homo sapiens calpain, small subunit 1 (CAPNS1), mRNA	NM_001749	Downregulated	-1.24	38
470	BF845798	Exonic	USP54	Homo sapiens ubiquitin specific peptidase 54, mRNA (cDNA clone IMAGE:6503621), with apparent retained intron	BC110845	Downregulated	-1.34	39
168	AW842748	Exonic	SIN3B	PREDICTED: Homo sapiens SIN3 homolog B, transcriptional regulator (yeast) (SIN3B), mRNA	XM_050561	Downregulated	-1.40	40
674	BF890754	Exonic	UQCRH	Homo sapiens ubiquinol-cytochrome c reductase hinge protein (UQCRH), mRNA	NM_006004	Downregulated	-1.56	41
682	AW603395	Exonic	YEATS4	Homo sapiens glioma-amplified sequence-41 (GAS41), mRNA	NM_006530	Downregulated	-1.55	42
873	CK327135	Exonic	N.A.	Homo sapiens cDNA FLJ41973 fis, clone SKNMC2004651	AK123967	Downregulated	-1.41	43
621	BF898656	Exonic	TRIP3	Homo sapiens thyroid hormone receptor interactor 3 (TRIP3), mRNA	NM_004773	Downregulated	-1.36	44
644	BQ314686	Exonic	MSH3	Homo sapiens mutS homolog 3 (E. coli) (MSH3), mRNA	NM_002439	Downregulated	-1.35	45
692	BF751539	Exonic	FOXK2	Homo sapiens forkhead box K2 (FOXK2), transcript variant 3, mRNA	NM_181431	Downregulated	-1.06	46
1002	BQ334482	Exonic	CDA	Homo sapiens cytidine deaminase (CDA), mRNA	NM_001785	Downregulated	-1.05	47
941	BE172353	Exonic	FUBP1	Homo sapiens far upstream element (FUSE) binding protein 1 (FUBP1), mRNA	NM_003902	Downregulated	-1.25	48
910	BE833259	Exonic	STN2	Homo sapiens mRNA for Stonin 2 variant protein	AB208948	Downregulated	-1.11	49
622	BE708661	Exonic	RXRG	Homo sapiens retinoid X receptor, gamma (RXRG), mRNA	NM_006917	Downregulated	-0.76	50

1809	BQ302418	Exonic	RAC1	Homo sapiens ras-related C3 botulinum toxin substrate 1 (rho family, small GTP binding protein Rac1) (RAC1), transcript variant Rac1, mRNA	NM_006908	Downregulated	-0.76	51
1302	BE163603	Exonic	TMEM141	Homo sapiens transmembrane protein 141 (TMEM141), mRNA	NM_032928	Downregulated	-0.60	52
2699	BF330679	Exonic	TMC4	Homo sapiens transmembrane channel-like 4 (TMC4), mRNA	NM_144686	Downregulated	-0.89	53
1460	BF856925	Exonic	WDR23	Homo sapiens WD repeat domain 23 (WDR23), transcript variant 2, mRNA	NM_181357	Downregulated	-0.78	54
1388	AW883439	Exonic	PRKAG1	Homo sapiens protein kinase, AMP-activated, gamma 1 non-catalytic subunit (PRKAG1), transcript variant 2, mRNA	NM_212461	Downregulated	-0.98	55
1402	AW883175	Exonic	CASP7	Homo sapiens caspase 7, apoptosis-related cysteine protease (CASP7), transcript variant delta, mRNA	NM_033338	Downregulated	-0.85	56
1469	AW365027	Exonic	CSNK1G2	Homo sapiens casein kinase 1, gamma 2 (CSNK1G2), mRNA	NM_001319	Downregulated	-0.91	57
1326	BF922962	Exonic	CTSB	Homo sapiens cathepsin B (CTSB), transcript variant 1, mRNA	NM_001908	Downregulated	-0.93	58
1458	AW880788	Exonic	LIMK1	Homo sapiens LIM domain kinase 1 (LIMK1), transcript variant dLIMK, mRNA	NM_016735	Downregulated	-0.90	59
1784	BF892704	Exonic	GSN	Homo sapiens gelsolin (amyloidosis, Finnish type) (GSN), transcript variant 2, mRNA	NM_198252	Downregulated	-0.61	60
704	BF089987	Exonic	NAG8	Homo sapiens nasopharyngeal carcinoma associated gene protein-8 (NAG8), mRNA	NM_014411	Downregulated	-0.90	61
2240	BG001664	Exonic	CD86	Homo sapiens CD86 antigen (CD28 antigen ligand 2, B7-2 antigen) (CD86), transcript variant 2, mRNA	NM_006889	Downregulated	-0.60	62
3327	BF082343	Exonic	LGALS8	Homo sapiens lectin, galactoside-binding, soluble, 8 (galectin 8) (LGALS8), transcript variant 3, mRNA	NM_201544	Downregulated	-0.56	63
2468	BF930663	Exonic	S100P	Homo sapiens S100 calcium binding protein P (S100P), mRNA	NM_005980	Upregulated	0.26	64
2003	BE087107	Exonic	N.A.	Homo sapiens cDNA: FLJ21288 fis, clone COL01927	AK024941	Upregulated	0.66	65
1770	BF922194	Exonic	N.A.	Homo sapiens KIAA0657 protein (KIAA0657), mRNA	XM_051017	Upregulated	0.55	66
1627	BE080712	Exonic	ZCCHC6	Homo sapiens zinc finger, CCHC domain containing 6 (ZCCHC6), mRNA	NM_024617	Upregulated	0.85	67
2226	AW852323	Exonic	N.A.	Homo sapiens cDNA FLJ45532 fis, clone BRTHA2030036	AK127440	Upregulated	0.64	68

1752	BF918989	Exonic	DNAJC10	Homo sapiens DnaJ (Hsp40) homolog, subfamily C, member 10 (DNAJC10), mRNA	NM_018981	Upregulated	0.72	69
1703	AW890141	Exonic	ITGAV	Homo sapiens integrin, alpha V (vitronectin receptor, alpha polypeptide, antigen CD51) (ITGAV), mRNA	NM_002210	Upregulated	0.81	70
3349	AW749339	Exonic	DACT1	Homo sapiens dapper homolog 1, antagonist of beta-catenin (xenopus) (DACT1), mRNA	NM_016651	Upregulated	0.90	71
3646	BF921541	Exonic	TERF2IP	Homo sapiens telomeric repeat binding factor 2, interacting protein (TERF2IP), mRNA	NM_018975	Upregulated	0.95	72
4021	BE927584	Exonic	GSPT1	Homo sapiens G1 to S phase transition 1 (GSPT1), mRNA	NM_002094	Upregulated	0.76	73
4462	BE711811	Exonic	TIAM1	Homo sapiens T-cell lymphoma invasion and metastasis 1 (TIAM1), mRNA	NM_003253	Upregulated	1.05	74
3635	BE708528	Exonic	PACSIN2	Homo sapiens protein kinase C and casein kinase substrate in neurons 2 (PACSIN2), mRNA	NM_007229	Upregulated	0.73	75
4143	BQ302049	Exonic	TRA1	Homo sapiens tumor rejection antigen (gp96) 1 (TRA1), mRNA	NM_003299	Upregulated	0.80	76
3351	BE163747	Exonic	KDELR2	Homo sapiens KDEL (Lys-Asp-Glu-Leu) endoplasmic reticulum protein retention receptor 2 (KDELR2), mRNA	NM_006854	Upregulated	1.15	77
3323	BE827259	Exonic	IDI1	Homo sapiens isopentenyl-diphosphate delta isomerase (IDI1), mRNA	NM_004508	Upregulated	0.83	78
3593	BF857286	Exonic	PICALM	Homo sapiens phosphatidylinositol binding clathrin assembly protein (PICALM), transcript variant 2, mRNA	NM_001008660	Upregulated	1.08	79
3525	BE087783	Exonic	HLCDGP1	Homo sapiens HLCDGP1 mRNA, complete cds	AF447582	Upregulated	1.10	80
1405	BG009544	Exonic	EGFR	Homo sapiens epidermal growth factor receptor (erythroblastic leukemia viral (v-erb-b) oncogene homolog, avian) (EGFR), transcript variant 1, mRNA	NM_005228	Upregulated	0.45	81
1799	BE074283	Exonic	PPM1D	Homo sapiens protein phosphatase 1D magnesium-dependent, delta isoform (PPM1D), mRNA	NM_003620	Upregulated	0.88	82
2202	BE831984	Exonic	SP140	Homo sapiens SP140 nuclear body protein (SP140), mRNA	NM_007237	Upregulated	0.91	83
2227	BQ292121	Exonic	RAB3B	Homo sapiens RAB3B, member RAS oncogene family (RAB3B), mRNA	NM_002867	Upregulated	0.80	84
4525	BE818647	Exonic	EFNA5	Homo sapiens ephrin-A5 (EFNA5), mRNA	NM_001962	Upregulated	0.81	85
2852	BF361046	Exonic	PECI	Homo sapiens peroxisomal D3,D2-enoyl-CoA isomerase (PECI), transcript variant 1, mRNA	NM_006117	Upregulated	1.09	86

2900	AW995233	Exonic	BMPR1A	Homo sapiens bone morphogenetic protein receptor, type IA (BMPR1A), mRNA	NM_004329	Upregulated	1.15	87
3741	BF736661	Exonic	FAT	Homo sapiens FAT tumor suppressor homolog 1 (Drosophila) (FAT), mRNA	NM_005245	Upregulated	1.34	88
594	AW994870	Exonic	PPP1CB	Homo sapiens protein phosphatase 1, catalytic subunit, beta isoform (PPP1CB), transcript variant 1, mRNA	NM_002709	Upregulated	0.33	89
287	BE833289	Exonic	VCL	Homo sapiens vinculin (VCL), transcript variant VCL, mRNA	NM_003373	Upregulated	0.30	90
954	BF757813	Exonic	PBEF1	Homo sapiens pre-B-cell colony enhancing factor 1 (PBEF1)	NM_005746	Upregulated	0.44	91
1314	BE703218	Exonic	MYOM2	Homo sapiens myomesin (M-protein) 2, 165kDa (MYOM2), mRNA	NM_003970	Upregulated	0.29	92
4484	AW604799	Exonic	S100A11	Homo sapiens S100 calcium binding protein A11 (calgizzarin) (S100A11), mRNA	NM_005620	Upregulated	0.77	93
2099	BE708316	Exonic	WIPI49	Homo sapiens WD40 repeat protein Interacting with phosphoInositides of 49kDa (WIPI49), mRNA	NM_017983	Upregulated	1.09	94
1566	BF848731	Exonic	MRPL14	Homo sapiens mitochondrial ribosomal protein L14 (MRPL14), nuclear gene encoding mitochondrial protein, mRNA	NM_032111	Upregulated	1.11	95
3137	BE939455	Exonic	ANKRD37	Homo sapiens low density lipoprotein receptor-related protein binding protein (Lrp2bp), mRNA	NM_181726	Upregulated	1.30	96
3755	BE768971	Exonic	MTAP	Homo sapiens methylthioadenosine phosphorylase (MTAP	NM_002451	Upregulated	1.00	97
3703	BQ334342	Exonic	LCP1	Homo sapiens lymphocyte cytosolic protein 1 (L-plastin) (LCP1), mRNA	NM_002298	Upregulated	1.48	98
3986	BF893393	Exonic	RNF149	Homo sapiens LOC389011 (LOC389011), mRNA.	XM_373998	Upregulated	0.90	99
3162	AW606115	Exonic	N.A.	Homo sapiens cDNA clone IMAGE:4752183	BC106022	Upregulated	0.69	100
3717	BE932430	Exonic	FOSL2	Homo sapiens FOS-like antigen 2 (FOSL2), mRNA	NM_005253	Upregulated	1.03	101
393	AW747998	Exonic	ZFYVE19	Homo sapiens zinc finger, FYVE domain containing 19 (ZFYVE19), mRNA	NM_032850	Upregulated	0.72	102
4338	AW805672	Exonic	N.A.	Homo sapiens cDNA FLJ38048 fis, clone CTONG2014264.	AK095367	Upregulated	0.61	103
4242	BF745581	Exonic	UNQ473	Homo sapiens DMC (UNQ473), mRNA	NM_198477	Upregulated	1.36	104

4239	BF332449	Exonic	MGEA5	Homo sapiens meningioma expressed antigen 5 (hyaluronidase) (MGEA5), mRNA	NM_012215	Upregulated	1.20	105
2897	BF743126	Exonic	CLK2	Homo sapiens CDC-like kinase 2 (CLK2), transcript variant 2, mRNA	NM_001291	Upregulated	0.99	106
3586	BF997724	Exonic	PAPPA	Homo sapiens pregnancy-associated plasma protein A, pappalysin 1 (PAPPA), mRNA.	NM_002581	Upregulated	0.63	107
1352	BE089538	Exonic	N.A.	Homo sapiens hypothetical protein MGC33365 (MGC33365), mRNA	NM_173552	Upregulated	0.85	108
2870	BF920836	Exonic	TFE3	Homo sapiens mRNA; cDNA DKFZp761J1810 (from clone DKFZp761J1810)	AL161985	Upregulated	0.96	109
3581	BF373987	Exonic	PTBP1	Homo sapiens polypyrimidine tract binding protein 1 (PTBP1), transcript variant 4, mRNA	NM_175847	Upregulated	1.11	110
3858	AW862460	Exonic	A2ML1	Homo sapiens alpha-2-macroglobulin-like 1 (A2ML1), mRNA	NM_144670	Upregulated	1.13	111
2605	BG002636	Exonic	HOXB6	Homo sapiens homeo box B6 (HOXB6), transcript variant 2, mRNA	NM_018952	Upregulated	1.24	112
4155	BG999796	Exonic	FES	Homo sapiens feline sarcoma oncogene (FES), mRNA	NM_002005	Upregulated	1.00	113
4237	BE089334	Exonic	N.A.	Homo sapiens mRNA; cDNA DKFZp762K067 (from clone DKFZp762K067)	CR627373	Upregulated	1.06	114
3674	BE694434	Exonic	AKAP11	Homo sapiens A kinase (PRKA) anchor protein 11 (AKAP11), transcript variant 1, mRNA	NM_016248	Upregulated	1.08	115
3705	BF895259	Exonic	CARD8	Homo sapiens caspase recruitment domain family, member 8 (CARD8), mRNA	NM_014959	Upregulated	1.14	116
3003	AW391808	Exonic	SIL	Homo sapiens TAL1 (SCL) interrupting locus (SIL), mRNA	NM_003035	Upregulated	1.30	117
4147	BE148898	Exonic	FANCE	Homo sapiens Fanconi anemia, complementation group E (FANCE), mRNA	NM_021922	Upregulated	1.56	118
3673	AW606595	Exonic	BTG1	Homo sapiens B-cell translocation gene 1, anti-proliferative (BTG1), mRNA	NM_001731	Upregulated	0.55	119
4099	BQ314772	Exonic	RAB1A	Homo sapiens RAB1A, member RAS oncogene family (RAB1A), mRNA	NM_004161	Upregulated	0.98	120
3339	BQ310838	Exonic	TRA1	Homo sapiens tumor rejection antigen (gp96) 1 (TRA1), mRNA	NM_003299	Upregulated	0.81	121
4358	BE163446	Exonic	SLC25A23	Homo sapiens solute carrier family 25 (mitochondrial carrier; phosphate carrier), member 23 (SLC25A23), mRNA	NM_024103	Upregulated	0.49	122

1924	BF742190	Exonic	N.A.	Homo sapiens cDNA FLJ14201 fis, clone NT2RP3002955	AK024263	Upregulated	0.49	123
2764	BF881091	Exonic	HDAC4	Homo sapiens histone deacetylase 4 (HDAC4), mRNA	NM_006037	Upregulated	0.54	124
2935	BG000772	Exonic	CD14	Homo sapiens CD14 antigen (CD14), mRNA	NM_000591	Upregulated	0.72	125
1833	BF919972	Exonic	N.A.	Homo sapiens neurotrophic tyrosine kinase, receptor, type 3 (NTRK3), mRNA	NM_002530	Upregulated	0.85	126
2007	BF805191	Exonic	NUDT16L1	Homo sapiens hypothetical protein MGC11275 (SDOS), mRNA	NM_032349	Upregulated	0.99	127
2956	BF371928	Exonic	TMEFF2	Homo sapiens transmembrane protein with EGF-like and two follistatin-like domains 2 (TMEFF2), mRNA	NM_016192	Upregulated	0.52	128
4402	BF853544	Exonic	S100A13	Homo sapiens S100 calcium binding protein A13 (S100A13), mRNA	XM_371380	Upregulated	0.79	129
2987	BF332165	Exonic	ALDH1A2	Homo sapiens aldehyde dehydrogenase 1 family, member A2 (ALDH1A2), transcript variant 2, mRNA	NM_170696	Upregulated	1.16	130
4513	BQ339716	Exonic	ERBB4	Homo sapiens v-erb-a erythroblastic leukemia viral oncogene homolog 4 (avian) (ERBB4), mRNA	NM_005235	Upregulated	0.89	131
983	AW352153	Exonic	PAWR	Homo sapiens PRKC, apoptosis, WT1, regulator (PAWR), mRNA	NM_002583	Upregulated	1.24	132
3908	BF996163	Exonic	N.A.	Homo sapiens hypothetical protein FLJ14490 (FLJ14490), mRNA	NM_032793	Upregulated	1.61	133
4495	BF926477	Exonic	IHPK2	Homo sapiens inositol hexaphosphate kinase 2 (IHPK2), mRNA	NM_016291	Upregulated	1.25	134
1835	CK326987	Exonic	ABCF1	Homo sapiens ATP-binding cassette, sub-family F (GCN20), member 1 (ABCF1), mRNA	NM_001090	Upregulated	0.99	135
1284	BG012009	Exonic	PHLDB2	Homo sapiens pleckstrin homology-like domain, family B, member 2 (PHLDB2), mRNA	NM_145753	Upregulated	1.13	136
3165	BF331880	Exonic	ARPC2	Homo sapiens actin related protein 2/3 complex, subunit 2, 34kDa (ARPC2), transcript variant 2, mRNA	NM_005731	Upregulated	1.51	137
2580	BF956989	Exonic	PALM2-AKAP2	Homo sapiens PALM2-AKAP2 protein (PALM2-AKAP2), transcript variant 1, mRNA	NM_007203	Upregulated	1.27	138
2098	CK326979	Exonic	RFC4	Homo sapiens replication factor C (activator 1) 4, 37kDa (RFC4), transcript variant 2, mRNA	NM_181573	Upregulated	1.64	139
4479	BF851788	Exonic	MTA1	Homo sapiens metastasis associated 1 (MTA1), mRNA	NM_004689	Upregulated	1.94	140

4009	BE815077	Exonic	CPA4	Homo sapiens carboxypeptidase A4 (CPA4), mRNA	NM_016352	Upregulated	1.77	141
3750	BE699157	Exonic	N.A.	Homo sapiens hypothetical protein DKFZp761G2113 (DKFZp761G2113), mRNA	XM_375456	Upregulated	1.72	142
2597	BF372924	Exonic	BITE	Homo sapiens p10-binding protein (BITE), mRNA	NM_024491	Upregulated	1.57	143
3117	BE156258	Exonic	EEF1A1	Homo sapiens eukaryotic translation elongation factor 1 alpha 1 (EEF1A1), mRNA	NM_001402	Upregulated	1.86	144
2960	BF365078	Exonic	ANXA6	Homo sapiens annexin A6 (ANXA6), transcript variant 2, mRNA	NM_004033	Upregulated	1.71	145
4435	AW369683	Exonic	ARHGAP26	Homo sapiens Rho GTPase activating protein 26 (ARHGAP26), mRNA	NM_015071	Upregulated	2.11	146
252	BF747659	Exonic	TCRG	Human T-cell receptor gamma chain VJCI-3(CII)-CIII region mRNA, 3' end.	M16804	Upregulated	1.39	147
928	BE841633	Exonic	OACT2	Homo sapiens O-acyltransferase (membrane bound) domain containing 2 (OACT2), mRNA	NM_138799	Upregulated	1.00	148
1028	BG002368	Exonic	LIFR	Homo sapiens leukemia inhibitory factor receptor (LIFR), mRNA	NM_002310	Upregulated	1.28	149
3773	BG008641	Exonic	HPGD	Homo sapiens hydroxyprostaglandin dehydrogenase 15-(NAD) (HPGD), mRNA	NM_000860	Upregulated	1.90	150
4431	BE166669	Exonic	B2M	Homo sapiens beta-2-microglobulin (B2M), mRNA	NM_004048	Upregulated	2.00	151
1558	AW860935	Exonic	LONRF1	Homo sapiens LON peptidase N-terminal domain and ring finger 1 (LONRF1), mRNA	NM_152271	Upregulated	1.71	152
2136	AW948647	Exonic	SOCS2	Homo sapiens suppressor of cytokine signaling 2 (SOCS2), mRNA	NM_003877	Upregulated	1.89	153
3730	AW373660	Exonic	SEPP1	Homo sapiens selenoprotein P, plasma, 1 (SEPP1), mRNA	NM_005410	Upregulated	1.94	154
2711	AW369662	Exonic	TMEPAI	Homo sapiens transmembrane, prostate androgen induced RNA (TMEPAI), transcript variant 4, mRNA	NM_199171	Upregulated	1.58	155
1718	BQ365807	Exonic	ELL2	Homo sapiens elongation factor, RNA polymerase II, 2 (ELL2), mRNA	NM_012081	Upregulated	1.68	156
2388	BF996314	Exonic	TMEPAI	Homo sapiens transmembrane, prostate androgen induced RNA (TMEPAI), transcript variant 4, mRNA	NM_199171	Upregulated	1.48	157
4497	BE769452	Exonic	ABCC4	Homo sapiens ATP-binding cassette, sub-family C (CFTR/MRP), member 4 (ABCC4), mRNA	NM_005845	Upregulated	1.68	158

4469	BF856737	Exonic	NKX3NO1	Homo sapiens NK3 transcription factor related, locus 1 (Drosophila) (NKX3-1), mRNA	NM_006167	Upregulated	1.94	159
2871	BE146184	Exonic	NDRG1	Homo sapiens N-myc downstream regulated gene 1 (NDRG1), mRNA.	NM_006096	Upregulated	2.45	160
2155	BF894880	Exonic	N.A.	Homo sapiens KIAA0056 protein (KIAA0056), mRNA	NM_015261	Upregulated	2.33	161
655	BF736834	Exonic	TMPRSS2	Homo sapiens transmembrane protease, serine 2 (TMPRSS2), mRNA	NM_005656	Upregulated	1.69	162
4403	BE814543	Exonic	EMP1	Homo sapiens epithelial membrane protein 1 (EMP1), mRNA	NM_001423	Upregulated	1.00	163
4262	BE702744	Exonic	N.A.	Homo sapiens hypothetical protein MGC27345, mRNA (cDNA clone MGC:27345 IMAGE:4670552), complete cds.	BC024231	Upregulated	2.33	164
2194	BQ367470	Exonic	CSF3R	Homo sapiens colony stimulating factor 3 receptor (granulocyte) (CSF3R), transcript variant 2, mRNA	NM_156038	Upregulated	2.65	165
2814	AW996919	Exonic	N.A.	Homo sapiens mRNA; cDNA DKFZp686L193 (from clone DKFZp686L193)	BX648778	Upregulated	2.47	166
1839	CK326989	Exonic	ZNF273	Homo sapiens zinc finger protein 273 (ZNF273), mRNA	NM_021148	Upregulated	2.39	167
2215	BE160926	Exonic	BFAR	Homo sapiens bifunctional apoptosis regulator (BFAR), mRNA	NM_016561	Upregulated	3.56	168
