# Supplementary material for: Androgen responsive intronic non-coding RNAs
Source: BMC Biol. 2007 Jan 30;5:4. doi: 10.1186/1741-7007-5-4 (PMC1800835; doi:10.1186/1741-7007-5-4)
Supplement: Additional File 4 — Supplementary Table 2. Androgen-responsive intronic transcripts. [file 1741-7007-5-4-S4.pdf]

Supplementary Table 2 - Androgen-responsive intronic transcripts.

39 intronic RNAs with significant expression changes in at least 3 consecutive time points were identified by two-class SAM analysis and clusterized hierarchicaly as described in *Methods.* Locus Name column indidates the protein-coding gene mapping in the same *locus* . Selection parameters were: fold-change ≥ 1.5 and false discovery rate (FDR) < 5%. The fold-change (log2) column reflects the relative abundance of signficantly up- and downregulated intronic RNAs in LNCap cells treated with androgen for 24 hours, as compared to untreated control cells. Selected RNAs in the same order from the hierarchical clustering shown in Figure 1 B.N.A. - Not available

Spot ID	GenBank Accession (Spotted EST)	Mapping	Locus Name	Locus Annotation	GenBank Accession (from Annotation)	Androgen effect	Fold-change (log2)	Hierarchical Clustering (order)
517	BF879099	Intronic	GAS6	Homo sapiens growth arrest-specific 6 (GAS6), mRNA	NM_000820	Downregulated	-1.70	1
156	BF360792	Intronic	RAB25	Homo sapiens RAB25, member RAS oncogene family (RAB25), mRNA	NM_020387	Downregulated	-1.27	2
70	CK327123	Intronic	SPIRE2	Homo sapiens spire homolog 2 (Drosophila) (SPIRE2), mRNA	XM_047462	Downregulated	-1.06	3
482	AW805354	Intronic	N.A.	Homo sapiens cDNA FLJ45018 fis, clone BRAWH3015610	AK126965	Downregulated	-0.50	4
559	BF854186	Intronic	PHC2	Homo sapiens partial mRNA for polyhomeotic 2 protein (PH2 gene)	AJ242730	Downregulated	-0.65	5
402	BF333281	Intronic	NIBP	Homo sapiens NIK and IKK{beta} binding protein (NIBP), mRNA	NM_031466	Downregulated	-1.24	6
790	AW860958	Intronic	DST	Homo sapiens dystonin (DST), transcript variant 1eA, mRNA	NM_015548	Downregulated	-1.03	7
1294	BF768459	Intronic	ACTN4	Homo sapiens actinin, alpha 4 (ACTN4), mRNA	NM_004924	Downregulated	-1.07	8
1293	BF368584	Intronic	DNAJC3	Homo sapiens DnaJ (Hsp40) homolog, subfamily C, member 3 (DNAJC3), mRNA	NM_006260	Downregulated	-1.13	9
1301	BF882783	Intronic	ACTN4	Homo sapiens actinin, alpha 4 (ACTN4), mRNA	NM_004924	Downregulated	-1.15	10
1592	AW815632	Intronic	CFLAR	Homo sapiens CASP8 and FADD-like apoptosis regulator (CFLAR), mRNA	NM_003879	Upregulated	0.48	11
105	CK327134	Intronic	RSNL2	Homo sapiens hypothetical protein FLJ21069 (FLJ21069), mRNA	NM_024692	Upregulated	0.19	12
2441	CK327184	Intronic	ITGA6	Homo sapiens integrin, alpha 6 (ITGA6), mRNA	NM_000210	Upregulated	0.22	13
3131	BE156190	Intronic	PALLD	Homo sapiens palladin, cytoskeletal associated protein (PALLD), mRNA	NM_016081	Upregulated	1.02	14

4255	BF332494	Intronic	SAP18	Homo sapiens sin3-associated polypeptide, 18kDa (SAP18), mRNA	NM_005870	Upregulated	1.13	15
2005	BE087892	Intronic	STARD13	Homo sapiens STARD domain containing 13, mRNA	BX647695	Upregulated	1.15	16
3479	BE061111	Intronic	TBN	Homo sapiens taube nuss homolog (mouse) (TBN), mRNA	NM_138572	Upregulated	1.36	17
2802	AW805635	Intronic	ERG	Homo sapiens v-ets erythroblastosis virus E26 oncogene like (avian) (ERG), transcript variant 2, mRNA	NM_004449	Upregulated	0.95	18
3912	BG004239	Intronic	NBPF9	Homo sapiens clone IIIa12 NBPF9 isoform 1 mRNA, complete cds, alternatively spliced.	AY894565	Upregulated	1.42	19
3115	BF350758	Intronic	KDELR2	Homo sapiens KDEL (Lys-Asp-Glu-Leu) endoplasmic reticulum protein retention receptor 2 (KDELR2), mRNA	NM_006854	Upregulated	0.97	20
2761	BF805741	Intronic	PMF1	Homo sapiens polyamine-modulated factor 1 (PMF1), mRNA	NM_007221	Upregulated	0.95	21
2821	CK327191	Intronic	PPP3CB	Homo sapiens protein phosphatase 3 (formerly 2B), catalytic subunit, beta isoform (calcineurin A beta) (PPP3CB), mRNA	NM_021132	Upregulated	0.99	22
3179	BF894911	Intronic	P2RX1	Homo sapiens purinergic receptor P2X, ligand-gated ion channel, 1 (P2RX1), mRNA	NM_002558	Upregulated	1.30	23
1200	AW880165	Intronic	TGFBR2	Homo sapiens transforming growth factor, beta receptor II (70/80kDa) (TGFBR2), mRNA	NM_003242	Upregulated	0.81	24
2786	AW805625	Intronic	N.A.	Homo sapiens hypothetical protein FLJ10154 (FLJ10154), mRNA	NM_018011	Upregulated	0.80	25
4357	CK327190	Intronic	ZNF644	Homo sapiens zinc finger motif enhancer binding protein 2 (Zep-2), transcript variant 3, mRNA	NM_032186	Upregulated	0.86	26
4360	BE168353	Intronic	PASK	Homo sapiens PAS domain containing serine/threonine kinase (PASK), mRNA	NM_015148	Upregulated	1.21	27
2758	BF848956	Intronic	MYO5A	Homo sapiens myosin VA (heavy polypeptide 12, myoxin) (MYO5A), mRNA	NM_000259	Upregulated	2.36	28
3483	BE062809	Intronic	UBE2V1	Homo sapiens ubiquitin-conjugating enzyme E2 variant 1 (Kua-UEV), transcript variant 1, mRNA	NM_199203	Upregulated	1.94	29
1296	BF768642	Intronic	N.A.	AGENCOURT_6594963 NIH_MGC_41 Homo sapiens cDNA clone IMAGE:5466041 5', mRNA sequence	BM811380	Upregulated	1.73	30
1283	CK327189	Intronic	P2RY14	Homo sapiens purinergic receptor P2Y, G-protein coupled, 14 (P2RY14), mRNA	NM_014879	Upregulated	1.26	31
814	AW819948	Intronic	PPP2R2A	Homo sapiens protein phosphatase 2 (formerly 2A), regulatory subunit B (PR 52), alpha isoform (PPP2R2A), mRNA	NM_002717	Upregulated	1.46	32
3900	AW819863	Intronic	PECAM1	Homo sapiens platelet/endothelial cell adhesion molecule (CD31 antigen) (PECAM1), mRNA	NM_000442	Upregulated	2.02	33
1690	BF926453	Intronic	XRCC1	Homo sapiens X-ray repair complementing defective repair in Chinese hamster cells 1 (XRCC1), mRNA	NM_006297	Upregulated	2.07	34

2458	BF926454	Intronic	ADD3	Homo sapiens adducin 3 (gamma) (ADD3), transcript variant 1, mRNA	NM_016824	Upregulated	2.26	35
1230	BF881469	Intronic	ALMS1	Homo sapiens Alstrom syndrome 1 (ALMS1), mRNA	NM_015120	Upregulated	1.82	36
4258	BF364131	Intronic	C16orf46	Homo sapiens hypothetical protein FLJ32702 (FLJ32702), mRNA	NM_152337	Upregulated	2.16	37
1629	CK327094	Intronic	ATF2	Homo sapiens activating transcription factor 2 (ATF2), mRNA	NM_001880	Upregulated	2.48	38
3503	BF364068	Intronic	MEG3	Homo sapiens hypothetical gene supported by BX161452 (LOC440199), mRNA	XR_000167	Upregulated	2.53	39
