# Supplementary material for: Androgen responsive intronic non-coding RNAs
Source: BMC Biol. 2007 Jan 30;5:4. doi: 10.1186/1741-7007-5-4 (PMC1800835; doi:10.1186/1741-7007-5-4)
Supplement: Additional File 5 — Supplementary Figure 3. In silico identification of ARE motifs in upstream regions of androgen-regulated intronic RNAs. [file 1741-7007-5-4-S5.pdf]

Supplementary Figure 3. ***In silico* identification of ARE motifs in upstream regions of androgen-regulated intronic RNAs.**

Genomic DNA sequence (3 kb) located at the 5' region upstream of the known transcriptional start site from each of the 39 androgen-regulated intronic RNAs identified by microarray analysis were searched for a motif corresponding to the ARE consensus sequence AGAACAnnnTGTTCT. GenBank Accession is given for the 24 genes where an ARE consensus motif was found. (Upper panel) ClustalW alignment using the 24 identified ARE motifs where the conserved residues are shown in black. (Lower panel) A consensus sequence displaying the frequency of each base proportional to the character height was generated by WEBLOGO [1]. This profile is comparable to the one observed by Nelson et al. when investigating ARE elements in upstream regions of protein-coding genes that are regulated by androgen in prostate cells [2].

GenBank Accession (EST)	Locus Name	ARE Sequence	Position	Genome Location	% Match
CK327134	**N A**	A G G A C A G A A T G T T T T	-2,056	**2p23.2**	83
BF333281	***NIBP***	A G A A G G T G C T G T T C A	-621	**8q24.3**	75
BF879099	***GAS6***	G G A A A A T C A T G T T C A	-1,528	**13q34**	75
AW860958	***DST***	A G A A C A T C T T T T T G C	-799	**6p12.1**	75
BF881469	***ALMS1***	G G A C C A G T T T T T T C T	-1,091	**2p13.1**	75
CK327189	***P2RY14***	A G A A C A G C A G G C T C T	-1,837	**3q25.1**	83
BF368584	***DNAJC3***	A G A A T A C C A T G T T G C	-1,608	**13q32.1**	75
BF768459	***ACTN4***	G G A A C A T G A A T T T C T	-785	**19q13.2**	75
BF768642	**N A**	T G A A C T G G G T T T T C T	+170	**Xq27.1**	75
BF882783	***ACTN4***	G G A A C C A C T G G T T C T	-1,839	**19q13.2**	75
CK327094	***ATF2***	T G A A C A G A C A C T T C T	-920	**2q31.1**	75
BE087892	***STARD13***	A G A T C A T G T G G T T C T	-2.239	**13q13.1**	83
CK327184	***ITGA6***	A G A A C A G G T T T T T G A	-2,788	**2q31.1**	75
BF926454	***ADD3***	T G A C C A T G C T G G T C T	-2,285	**10q25.1**	75
BF848956	***MYO5A***	A G T A C A T A A T T T A C T	-812	**15q21.2**	75
BF805741	***PMF1***	A G A A A T T C T T G C T C T	-2,104	**1q22**	75
AW805635	***ERG***	A G A A C T G C T T T G T C T	+246	**21q22.2**	75
BF350758	***KDELR2***	A G A A C A G C T G C T T C T	+219	**7p22.1**	83
BE156190	**N A**	A G A C C A G T T T T A T C T	-181	**4q32.3**	75
BE062809	***UBE2V1***	A G A A C A A F A T G T T A A	-2.786	**20q13.13**	83
AW819863	***PPP2R2A***	A G A G C A G G A A G C T C T	-881	**17q23.3**	75
BF332494	***SAP18***	A G A C C A G C C T G G T C A	-414	**13q12.11**	75
BF364131	**N A**	T G A C C A G G C T G G T C T	-2,481	**16q23.2**	75
CK327190	***ZNF644***	A G A A T C T A C T G T T C C	-283	**1p22.2**	75

ARE Consensus Sequence

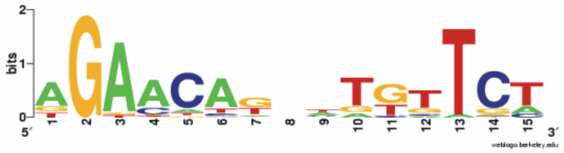

1. Crooks GE, Hon G, Chandonia JM, Brenner SE: **WebLogo: a sequence logo generator**. *Genome Res* 2004, **14**:1188-1190.
2. Nelson PS, Clegg N, Arnold H, Ferguson C, Bonham M, White J, Hood L, Lin B: **The program of androgen-responsive genes in neoplastic prostate epithelium**. *Proc Natl Acad Sci U S A* 2002, **99**:11890-11895.
