# Supplementary material for: Androgen responsive intronic non-coding RNAs
Source: BMC Biol. 2007 Jan 30;5:4. doi: 10.1186/1741-7007-5-4 (PMC1800835; doi:10.1186/1741-7007-5-4)
Supplement: Additional File 6 — MIAME file. [file 1741-7007-5-4-S6.pdf]

Supplementary information for the manuscript by Louro et al., 2006.

The MIAME Checklist (http://www.mged.org/Workgroups/MIAME/miame_checklist.html)

1. Experiment Design:

1.1. Type of experiment:

The experiments described here aim to generate a set of temporal expression profiles in a prostate tumor cell line treated with androgen, using a custom-designed cDNA microarray platform with 4,608 unique elements in replicate (9,216) enriched in gene fragments that map to intronic regions of known human genes. The platform consists of 3,355 probes of human cDNA fragments that map in the human genome sequence either to intronic regions of known genes (822), to unannotated regions of the human genome (241) or to known exons of RefSeq genes (2,292). We compared the expression profiles obtained from androgen treated cells with that of control samples to evaluate the androgen-modulated transcriptional activity from intronic regions.

1.2. Experimental factors:

Prostate cancer cells LNCaP were cultured for 24 hours in androgen-deprived medium, RPMI 1640 with 5% (wt/vol) charcoal-stripped FCS (Life Technologies). 1 nM of the synthetic androgen R1881 (NEN - Life Sciences Products) or ethanol vehicle control was added to fresh RPMI 1640 medium, with 10% (wt/vol) charcoal-stripped FCS. Cells were harvested for RNA isolation at several time points: 0, 6, 9, 12, 18, 24, and 48 hours.

1.3. The number of hybridizations performed in the experiment.

For each time point, RNA from two biological replicates of androgen-treated or untreated control cells was isolated and subsequently used for microarray hybridizations. Each biological replicate from each time point

was hybridized once to a microarray, performing a total of 26 hybridizations.

1.4. The type of reference used for the hybridizations, if any.

All hybridizations included external mRNA spikes for quality control of the experiments (detailed below in section 2.5) and 15 µg of total RNA from a reference pool containing equal amounts of RNA from three prostate cancer cell lines: LNCaP, DU145 and PC3.

1.5. Hybridization design:

For each time point, RNA aliquots from androgen or ethanol treated cells (15 µg) were used to generate cDNA targets labeled with Cy5-dUTP by reverse transcription. In parallel, RNA aliquots from the reference pool were used to generate Cy3-dUTP labeled targets. Labeled targets from each experimental point and from the reference RNA pool were combined and hybridized to microarrays using an Automated Slide Processor (Amershan Biosciences). Two hybridizations were performed for each time point, each using RNA from an independent biological replicate.

1.6. Quality control steps taken:

Integrity of total RNA was checked by electrophoresis using the Bio Sizing total RNA Nano assay in the 2100 Bioanalyzer (Agilent Technologies).

Two replicate hybridization experiments were performed for each time point sample, as describe in the previous section.

2. Samples used, extract preparation and labeling:

2.1. The origin of the biological sample

Prostate carcinoma cell lines LNCaP, DU145 and PC3 were obtained from ATCC and maintained using the suggested medium supplemented with 10% (vol/vol) fetal calf serum (FCS), 3 mM L-glutamine, 100 µg/ml

streptomicine and 100 U/ml peniciline. LNCaP cells were grown in RPMI 1640 whereas DU145 and PC3 cells in DMEM.

2.2. Manipulation of biological samples and protocols used:

All samples were harvested and washed with PBS prior to RNA extraction.

2.3. Protocol for preparing the hybridization extract:

Total RNA was purified from experimental and control cells by CsCl gradient, after cell lysis (4M guanidine isotiocianate; 0.1M b-mercaptoethanol; 25 mM sodium citrate pH 7.0). Total RNA (100 µg sample) was treated with of RNAse-free DNAse (RNeasy – Qiagen) for 15min to minimize genomic DNA contaminants. Purity of the isolated RNA was estimated by measuring the ratio A 260/A 280. Integrity of total RNA was checked by electrophoresis using the Bio Sizing total RNA Nano assay in the 2100 Bioanalyzer (Agilent Technologies).

2.4. Labeling protocol:

Labeled targets for hybridizations were generated from total RNA in reverse transcription reactions using oligo dT as RT primer, following strictly the protocol accompanying the CyScribe First-Strand labeling kit (Amersham Biosciences, Piscataway, NJ). In short, 15 µg total RNA was combined with primers in a total volume of 11 µl, denatured at 70°C for 10 minutes, put on ice for 30 seconds, spin down and placed at room temperature for 10 minutes. Nine microliters of a premix solution was added containing 5X Superscript II reaction buffer (5X), 200 U Superscript II reverse transcriptase, 0.1 M DTT, dNTP mix (2 mM dATP, 2 mM dGTP, 2 mMdTTP ,1 mM dCTP) and 1 mM of either Cy3- or Cy5-dCTP. After addition of the premix, the reaction was kept at room temperature for 10 minutes and then incubated at 42°C for 1.5 hr. RNA templates were removed by alkaline hydrolysis by adding 2 µl of 2.5 M sodium hydroxide and incubating the mixture at 37° C for 15 minutes. The labeling reaction was neutralized with 10 µl 2 M MOPS free acid and labeled targets were

purified using 96-well Millipore Multiscreen filter plates as follows: 5 volumes of 5.3 M Guanidine-HCl: 150 mM KOAc were added to labeling reactions. The mixture was applied on the plate and washed 4X with 80% EtOH by centrifugation at 3500 rpm for 5 min. Residual EtOH was spin out by an additional centrifugation at 3500 rpm for 5 minutes. Labeled targets were eluted in 50 μl 10mM Tris pH 8.5, by spinning at 3000 rpm for 5 minutes, dried on a SpeedVac and kept at -20°C, protected from light until use.

2.5. External controls (spikes).

In each labeling reaction we added 2.5 μl of a control mRNA mix from the Lucidea Microarray ScoreCard kit (Molecular Dynamics). This spike mRNA mix is complementary to a set of 32 yeast intergenic sequences that were deposited in the microarray and do not cross-hybridize with human labeled cDNAs.

3. Hybridization procedures and parameters:

Labeled targets were ressuspended in 250 μl of 1 x hybridization buffer (25% formamide, 12.5% of proprietary Microarray Hybridization Buffer Version 2 from Amersham Biosciences cat. RPK0325) denatured for 2 min at 92 ºC and centrifuged at 13,000 rpm for 5 min.

All slide processing steps (blocking, hybridization, washing) were carried out on an automated slide processor (ASP) from Amersham Biosciences. The slides were incubated for 16h at 42ºC and subsequently washed at room temperature in 1xSSC/0.2%SDS for 5min, 0.1xSSC/0.2%SDS for 5 min and in 0.1XSSC for 3 min. After the washing steps, slides were flushed with isopropanol and dried with an air flush at 42ºC.

4. Measurement data and specifications:

4.1. Type of scanning hardware and software used:

Images were obtained from each channel by laser scanning using GenePix 4000B microarray scanner (Axon Instruments). Slides were scanned with the following parameters:

- scanning resolution: 10-μm pixel
- excitation wave length: 532 nm (for Cy3-labeled targets) or 633 nm (for Cy5-labeled targets)
- emission filters: 575 nm (for Cy3-labeled targets) or 675 nm (for Cy5-labeled targets).
- PMT voltage: 350 volts (for Cy3-labeled targets) and 600 volts (for Cy5-labeled targets).

4.2. Type of image analysis software used

Raw images were analyzed using the ArrayVision software (Version 8.0, Imaging Research Inc.). An array template (or grid) was first automatically aligned to locate the position of each spot in the array, and subsequently manually adjusted to obtain the best possible alignment.

4.3. The quantization based on the images.

We employed in our analyses the Artifact-Removed density value (ARM) calculated for each spot by ArrayVision. The ARM value represents the average of all the pixels remaining in the spot, after first removing pixels with density values that exceed four median absolute deviations (MADs) from the median. This procedure removes the influence of image artifacts (e.g., dust particles) on density estimation. The ARM density for each spot was subtracted by the median background surrounding the spot (distance of 2 pixels with 3 widths of pixels).

Spot quantization matrixes (the “raw” output of the image analysis software) were obtained from all hybridizations. Measurements correspond to the raw data generated by the Image analysis software. We have consolidated in a single file all measurements obtained in replicate hybridizations in order to facilitate subsequent normalization and averaging of replicated spots.

4.4. Data selection and transformation procedures.

As each microarray contains two replicates of each spotted cDNA, a total of four replicate measurements were obtained for each spot in each time point. The raw data of each spot was compared to the value of hibridization background using the Lucidea Microarray Scorecard to determine which spots have a signal above or below the detection limit of each hybridization, where the background was given by the signal measured on a negative control (plant cDNA). If their signal were at least 3 standard deviations above the average signal of the negative control, spots were considered expressed in LNCaP cells. We decided to use the mean intensity signal (40% trimmed) of each dataset for normalization between experiments, and to calculate the treatment to control sample ratios using only the Cy5-labelled sample measurements.

Spots with statistically significant expression changes in at least 3 consecutive time points in response to androgen stimulation were identified using the Significance Analysis of Microarray data (SAM) approach, using as parameters: two-class response (paired data), 1000 permutations, K-Nearest Neighbors Imputer, fold-change ≥ 1.5 and false discovery rate (FDR) < 5%. Replicate spots were averaged for further analysis.

5. Array Design

The microarray platform used in this project (Generation-III array spotter - Molecular Dynamics) deploys up to 4,608 distinct cDNA spots in duplicate per glass slide, comprising a total of 9,216 elements per array. We selected among the clones generated in the HCGP project (http://www.ludwig.org.br/ORESTES/) a set of 1,301 ORESTES with no similarity to coding sequences of full-length human transcripts ("no-match" sequences). An additional set of 2,385 annotated ORESTES clones were included in the microarray, based on a list of cancer-related genes. The remaining 922 spots in the slide were reserved to contain replicate sets of housekeeping genes, as well as positive and negative control cDNAs. A set

of 32 cDNAs from the Lucidea Microarray ScoreCard (Molecular Dynamics) was included in the microarray. In addition to positive (complex DNA, housekeeping genes) and negative (plant cDNA) controls, the ScoreCard kit comprises a set of intergenic yeast DNA fragments that do not hybridize with human labeled cDNA, and a complementary mRNA mix that is spiked in the labeling reactions. The well-defined amount of exogenous mRNA present in the spike mixes was used to monitor the quality of the hybridizations, and the plant negative controls were employed to detect which clones were expressed above or below the detection limit of the hybridization.

Reporter molecules deposited in the microarray consist of cDNA fragments resulting from PCR amplification of ORESTES cDNA clones (Dias-Neto *et al.*, 2000). These were arranged in 12 sub-arrays containing each one 12 rows and 32 columns of spots (384 spots/sub-array). Rows 1 to 5 comprise no-match human cDNAs. Rows 6 to 10 comprise cDNAs from known human genes. Rows 11 and 12 of each sub-array contain the 32 ScoreCard control cDNAs and 32 prostate expressed control cDNAs plus housekeeping cDNAs, respectively.

Each reporter is unambiguously identified in the array by the Spot Label (ID), which indicates the position of the spot along the array. Each reporter is also identified by GenBank accession for the EST (GB_ACC) from which it derived. In addition, there are columns indicating the annotation of the EST based on searches against Reference Sequences (RefSeq annotation - 2005), as well as the GenBank accession number for this sequence (RefSeq – 2005).

6.1 <u>The method of reporter preparation.</u>

We used template cDNA clones that were generated and sequenced in the Human Cancer Genome Project (Camargo *et al.*, 2001). The clone collection is stored frozen in bar-coded 96-well plates, and selected clones were re-arrayed with an automated robotic operation. cDNA fragments were generated from these clones by PCR amplification with universal primers. These PCR products were purified by filtration on Multiscreen plates (Millipore cat. # MAFB NOB 50) and an aliquot was used to determine the size of the

product and to quantify the amount of cDNA recovered. This was done by agarose gel electrophoresis stained with ethydium bromide and scanned on the ImageMaster Video Digital System (Amersham Pharmacia). cDNA samples were diluted 1:1 in DMSO and spotted at high density into silane-coated, reflective Type-7 glass slides (Molecular Dynamics) using a GenIII microarray spotter robot (Molecular Dynamics). Following spotting, cDNAs were fixed to slide surface by UV cross-linking (500 mJ) and kept on a low humidity environment until use.

MIAME checklist references:

Camargo AA, Samaia HP, Dias-Neto E, Simao DF, Migotto IA, Briones MR, Costa FF, Nagai MA, Verjovski-Almeida S, Zago MA, Andrade LE, Carrer H, El-Dorry HF, Espreafico EM, Habr-Gama A, Giannella-Neto D, Goldman GH, Gruber A, Hackel C, Kimura ET*, et al.* (2001). *Proc. Natl. Acad. Sci. USA*, **98,** 12103-12108.

Dias-Neto E, Correa RG, Verjovski-Almeida S, Briones MR, Nagai MA, da Silva W, Jr., Zago MA, Bordin S, Costa FF, Goldman GH, Carvalho AF, Matsukuma A, Baia GS, Simpson DH, Brunstein A, de Oliveira PS, Bucher P, Jongeneel CV, O'Hare MJ, Soares F*, et al.* (2000). *Proc. Natl. Acad. Sci. USA*, **97,** 3491-3496.
